# Specific Position of Halogen in Crystalline TADF Scintillators Enables Efficient Triplet Harvesting Through Vibrational Modulation of Spin–Orbit Coupling

**DOI:** 10.1002/adma.74520

**Published:** 2026-08-06

**Authors:** Illia E. Serdiuk, Michał Mońka, Artur Sikorski, Konrad J. Drozdowski, Mohanad S. Eid, Krzysztof Wisniewski, Damian Trzybiński, Marcin E. Witkowski, Winicjusz Drozdowski

**Affiliations:** ^1^ Faculty of Mathematics, Physics and Informatics University of Gdańsk Gdańsk Poland; ^2^ Faculty of Chemistry University of Gdansk Gdańsk Poland; ^3^ Faculty of Physics, Astronomy and Informatics Institute of Physics Nicolaus Copernicus University in Toruń Toruń Poland; ^4^ Biological and Chemical Research Centre University of Warsaw Warsaw Poland

**Keywords:** gamma spectroscopy, heavy‐atom effect, intersystem crossing, organic crystal, organic scintillator, photoluminescence, spin‐orbit coupling, thermally activated delayed fluorescence, radioluminescence, X‐ray imaging

## Abstract

Thermally activated delayed fluorescence (TADF) offers a powerful route for harvesting triplet excitons in organic scintillators, yet achieving simultaneously small singlet–triplet gaps and efficient spin–orbit coupling (SOC) in rigid molecular crystals remains a fundamental challenge. Here we demonstrate that targeted halogen substitution can activate vibrationally assisted spin‐flip channels that dramatically enhance triplet harvesting in crystalline donor‐acceptor emitters, tailoring them for scintillators with higher light yield and faster response times. Using **DMAC‐TRZ** derivatives bearing fluorine or chlorine substituents, we combine single‐crystal structural analysis, temperature‐resolved photoluminescence, radioluminescence spectroscopy, and quantum‐chemical calculations to reveal how subtle changes in halogen chemistry control excited‐state dynamics. Fluorination lowers the rISC activation barrier to 7.1 meV, consistent with an almost degenerate emissive singlet–triplet manifold, whereas chlorine additionally introduces dynamic SOC enhancement mediated by Cl‐atom vibrations within the crystal lattice. As a result, the chlorinated crystal exhibits an exceptionally small *E*_a_ of 3.9 meV, (sub)microsecond‐scale delayed fluorescence, and a scintillation yield of 26 000 photons MeV^−1^. These results reveal a powerful, targeted approach to achieving near S_1_‐T_1_ degeneracy combined with vibrationally activated heavy‐atom effects, enabling high‐performance TADF scintillators in organic crystals.

## Graphical abstract

Specific *ortho*‐halogenation transforms crystalline TADF emitters into efficient organic scintillators. Fluorine reduces the effective singlet–triplet separation, while chlorine combines near‐degenerate excited states with vibrational modulation of spin–orbit coupling. This targeted design enables rapid triplet harvesting, bright radioluminescence, and excellent operational stability suitable for x‐ray imaging.

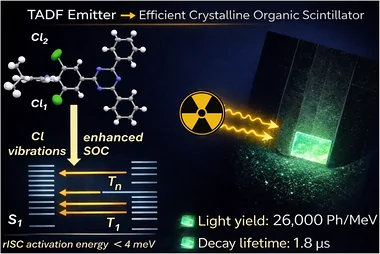

## 1 Introduction

Organic scintillators are evolving rapidly through multiple strategies. Traditional approaches include fluorescent organic molecules embedded in polymer matrices, aggregation‐induced emission (AIE) systems, and room‐temperature phosphorescence (RTP) materials. In conventional fluorescent systems, only singlet excitons contribute to light emission, leaving the 75% triplet population unused and thereby limiting internal quantum efficiency. RTP‐based materials can harvest triplets but often suffer from long emission lifetimes and oxygen sensitivity, resulting in poor timing resolution and operational instability.

Organic light‐emitting and scintillating systems based on thermally activated delayed fluorescence (TADF) emitters have gained considerable attention over the past decade owing to their unique ability to harvest both singlet and triplet excitons via reverse intersystem crossing (rISC, Figure 1A), thereby enabling nearly 100% internal quantum efficiency (IQE). Fast and efficient upconversion of triplet excitons to singlets are both critical for high light yield and precise timing in radiation detection via the TADF mechanism [1, 2, 3].

**FIGURE 1:**
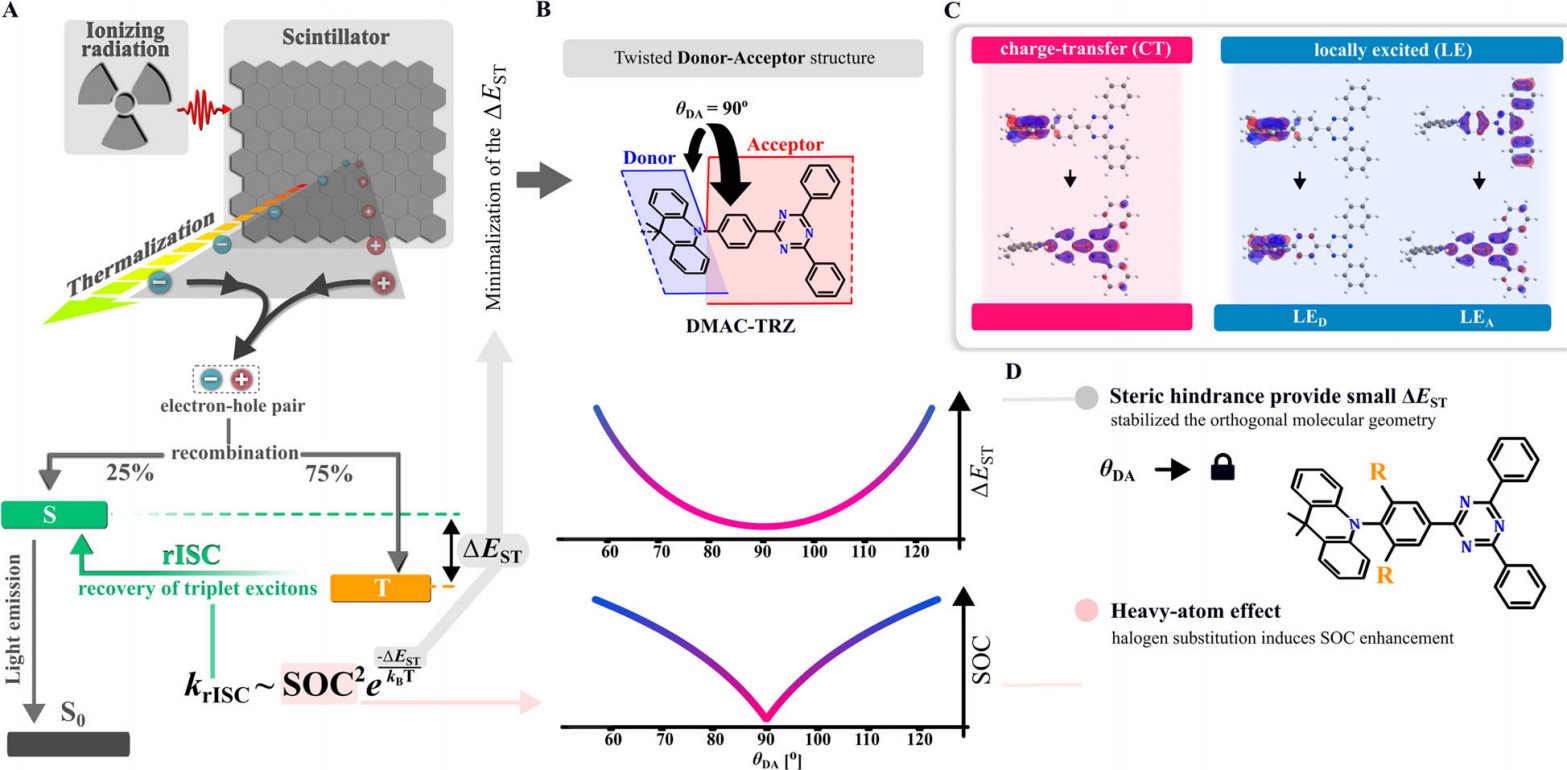
(A) Schematic illustration of the TADF mechanism in scintillation. (B) Donor‐acceptor molecular structure exemplified by the **DMAC‐TRZ** emitter, correlation between the D‐A dihedral angle (*θ*_DA_) with Δ*E*_ST_ and SOC. (C) Molecular orbitals involved in the key electronic excited states. (D) Molecular design concept for Δ*E*_ST_ reduction and SOC enhancement in this work.

In the OLED field, current all‐organic TADF emitters now achieve external quantum efficiencies above 30%, comparable to those of heavy‐metal organic complexes [4, 5, 6]. In parallel, organic scintillators are rapidly evolving from conventional bulk materials toward molecular crystals, polymeric systems, and flexible films for radiation detection and x‐ray imaging [7, 8, 9]. Within this broader development, several TADF‐based scintillators have demonstrated excellent radioluminescence (RL) performance under ionizing radiation [10]. Although this field remains relatively young, some TADF scintillators have been reported to exhibit promising scintillation performance under x‐ray excitation [1], although the absolute light‐yield values reported in the literature vary considerably depending on the measurement methodology and remain a subject of ongoing discussion.

Despite these achievements, the increase of efficiency and stability, particularly through shortening the delayed fluorescence lifetime (*τ*_DF_) to sub‐microsecond timescales, remains a major challenge. Addressing this requires bridging molecular‐scale structural and vibrational control with collective excited‐state dynamics that determine macroscopic scintillation performance. As in OLED applications, the key photophysical parameter to improve TADF scintillator performance is the rISC rate, which directly depends on two main factors: the singlet‐triplet energy gap (Δ*E*_ST_) and the spin‐orbit coupling constant (SOC). An ideal, fast‐rISC scintillator should combine minimal Δ*E*_ST_ to facilitate thermal upconversion and maximal SOC to enable a high probability of spin‐flip between excited states.

The molecular design strategies enabling simultaneous minimization of Δ*E*_ST_ and increase of SOC are very rare [11]. Δ*E*_ST_ is largely governed by the separation between the frontier molecular orbitals. In donor‐acceptor (D‐A) emitters, a small Δ*E*_ST_ is typically achieved by maintaining the dihedral angle (*θ*_DA_) between the donor and acceptor fragments close to 90°. However, such an orthogonal arrangement is unfavorable for spin–orbit coupling between *S*_1_ and *T*_1_, decreasing ⟨*S*_1_∣Ĥ_SO_∣*T*_1_⟩ to zero. This is due to the fact that SOC depends on the change in orbital momentum, which vanishes for the *S*_1_ and *T*_1_ states of the same charge‐transfer (CT) character. In light‐atom materials, SOC can be enhanced by molecular vibrations that induce deviations of *θ*_DA_ from 90°, or more effectively by mixing CT and locally excited (LE) states within the triplet manifold (Figure 1B,C) [12]. The role of specific atomic vibrations both in minimizing ΔE_ST_ and in activating SOC within rigid environments has only recently been recognized as a key factor governing efficient TADF [13, 14].

The strategic, vibrationally‐targeted introduction of abundant heavy atoms (Cl, S, P, Br, Se, I) represents a particularly promising approach for the design of molecular scintillators as it increases the probability of absorption of ionizing radiation and thus enhances detection efficiency [15]. From a photophysical standpoint, heavy atoms enhance the existing or open additional SOC channels that facilitate spin‐flip processes. However, in organic systems, their impact remains poorly understood due to the complex interplay between electronic coupling, molecular conformation, and intermolecular interactions, altering molecular packing, local polarity, and structural stability. Recent studies have revealed that peripheral halogen atoms (Cl, Br) enhance SOC not only through their intrinsic SOC but also via dynamic heavy‐atom effects, where atomic motion modulates frontier orbital overlap [16, 17]. The spatial alignment of these atoms is also critical: depending on their position, they may interact with multiple molecular fragments, leading either to self‐compensating or synergistically enhanced SOC [18]. In condensed phases, such as crystals, plastics, or films, *intermolecular contacts involving heavy atoms* can further amplify or suppress SOC, making their orientation and dynamic accessibility key parameters for photophysical optimization [19]. Importantly, the magnitude of various heavy‐atom effects is strongly conformation‐dependent and is likely activated by specific *molecular vibrations involving heavy atoms* [20].

In this context, we have recently suggested a single‐atom substitution strategy that enables precise control over excited‐state dynamics by replacing hydrogen with fluorine or chlorine within the donor–acceptor scaffold [10]. Positioning a moderate heavy atom like chlorine in the *ortho* position to the donor fragment markedly enhances the rISC rate, reduces the TADF lifetime, indicating improved triplet harvesting.

Unlike previous approaches that primarily exploit halogenation for the increase of x‐ray attenuation [2, 8], we propose here a strategy focused primarily on the modulation of molecular photophysics and triplet harvesting. Electronically similar F and Cl atoms with different atomic masses are placed at the *ortho* positions of the D‐A linkage, where they directly control *θ*_DA_ and help to differentiate structural and heavy‐atom contributions to rISC and SOC. In the Cl derivative, this same modification combines a very small rISC activation energy with vibrationally accessible SOC channels. In the crystalline derivatives of **DMAC‐TRZ** investigated here, namely **DMAC‐diF‐TRZ** and **DMAC‐diCl‐TRZ** (Figures 1D and 2), we apply this concept to a structurally defined crystal environment. Notably, the steric and electronic effects of chlorine substitution not only stabilized the orthogonal molecular geometry but also promoted the formation of large, well‐faceted crystalline domains with nearly cubic morphology, which is an important feature for high‐efficiency radioluminescence applications.

**FIGURE 2:**
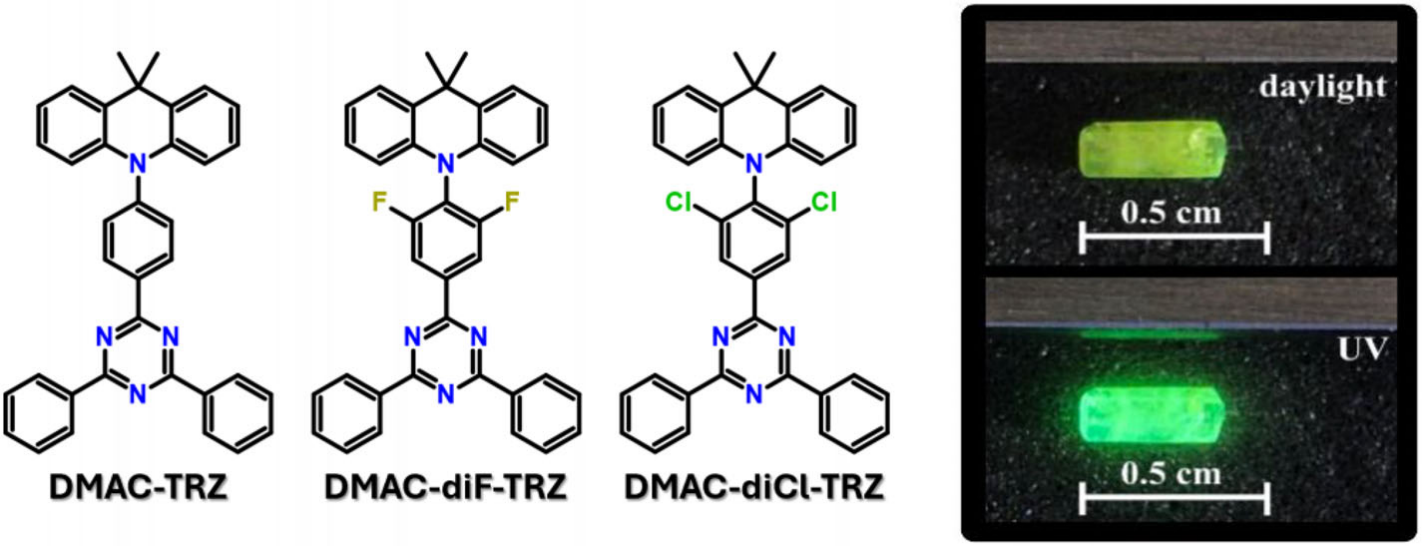
Chemical structures of the studied compounds and photographs of the **DMAC‐diCl‐TRZ** crystal under daylight and UV illumination.

Finally, our specific focus lies on the crystalline phase, which provides a structurally well‐defined platform to correlate molecular geometry, packing, and excitonic coupling with triplet‐harvesting dynamics. It should be noted that direct pulse height measurements in organic materials are notoriously challenging due to their low density, long decay times, and the difficulty of growing large, defect‐free single crystals. As a result, reported light yield values are typically based on relative spectral comparisons and should be regarded as estimates of x‐ray‐to‐light conversion efficiency rather than absolute scintillation yield. As direct comparison with inorganic benchmarks must therefore be made with caution, we aim here to provide a more accurate assessment of radioluminescence efficiency in state‐of‐the‐art TADF‐based organic scintillators.

## 2 Results and Discussion

### 2.1 Synthesis

To synthesize **DMAC‐diF‐TRZ,** we took advantage of the different reactivity of C‐F bonds at *para* and *meta* positions relative to the *s*‐triazine acceptor (Scheme S1). First, 3,4,5‐trifluorophenylboronic acid was cross‐coupled with 2‐(4‐chlorophenyl)‐4,6‐diphenyl‐*s*‐triazine under Suzuki‐Miyaura conditions to afford the trifluorinated TRZ intermediate with an almost quantitative yield (99%). The final S_N_Ar reaction between this intermediate and deprotonated 9,9‐dimethyl‐9,10‐dihydroacridine (DMAC) selectively displaced the fluorine atom at *para* position, giving **DMAC‐diF‐TRZ** in 87% yield.

**DMAC‐diCl‐TRZ** was synthesized through a three‐step route starting from DMAC and 2,4‐dichloro‐5‐bromofluorobenzene. In the first step, DMAC was introduced via an S_N_Ar reaction with NaH in DMF at 150°C, affording the aryl‐DMAC intermediate in 53% yield. Subsequent Miyaura borylation using bis(pinacolato)diboron and PdCl_2_(dppf) gave the corresponding boronate ester in 87% yield, which was then coupled with 2‐(4‐chlorophenyl)‐4,6‐diphenyl‐*s*‐triazine under Suzuki‐Miyaura conditions to yield the final product in 67% yield. The overall yield of **DMAC‐diCl‐TRZ** was approximately 31%.

### 2.2 Single‐Crystal X‐Ray Diffraction Analysis

#### 2.2.1 Molecular Geometry

The dihedral angle between the donor and acceptor units, as well as the planarity of the donor, are known to play a key role in determining luminescence properties and governing spin‐flip dynamics. The dihedral angles between DMAC and the phenylene linker (*θ*_DA_) is similar in all three compounds, with values of 88.3°, 86.1°, and 87.0° for **DMAC‐TRZ** [21], **DMAC‐diF‐TRZ**, and **DMAC‐diCl‐TRZ**, respectively (Figure S1). However, the planarity of DMAC shows a pronounced dependence on the substituent. Both **DMAC‐TRZ** and, even more distinctly, **DMAC‐diF‐TRZ** exhibit a bent (butterfly‐like) geometry of the DMAC donor fragment, with angles between the mean planes defined by the right‐ and left‐hand halves of the acridine skeleton of 10.2° and 29.7°, respectively (Figure 3A). In contrast, **DMAC‐diCl‐TRZ** displays a much more planar DMAC structure, with the corresponding interplanar angle reduced to 5.4°, confirming that chlorine substitution effectively locks the donor into a rigid conformation.

**FIGURE 3:**
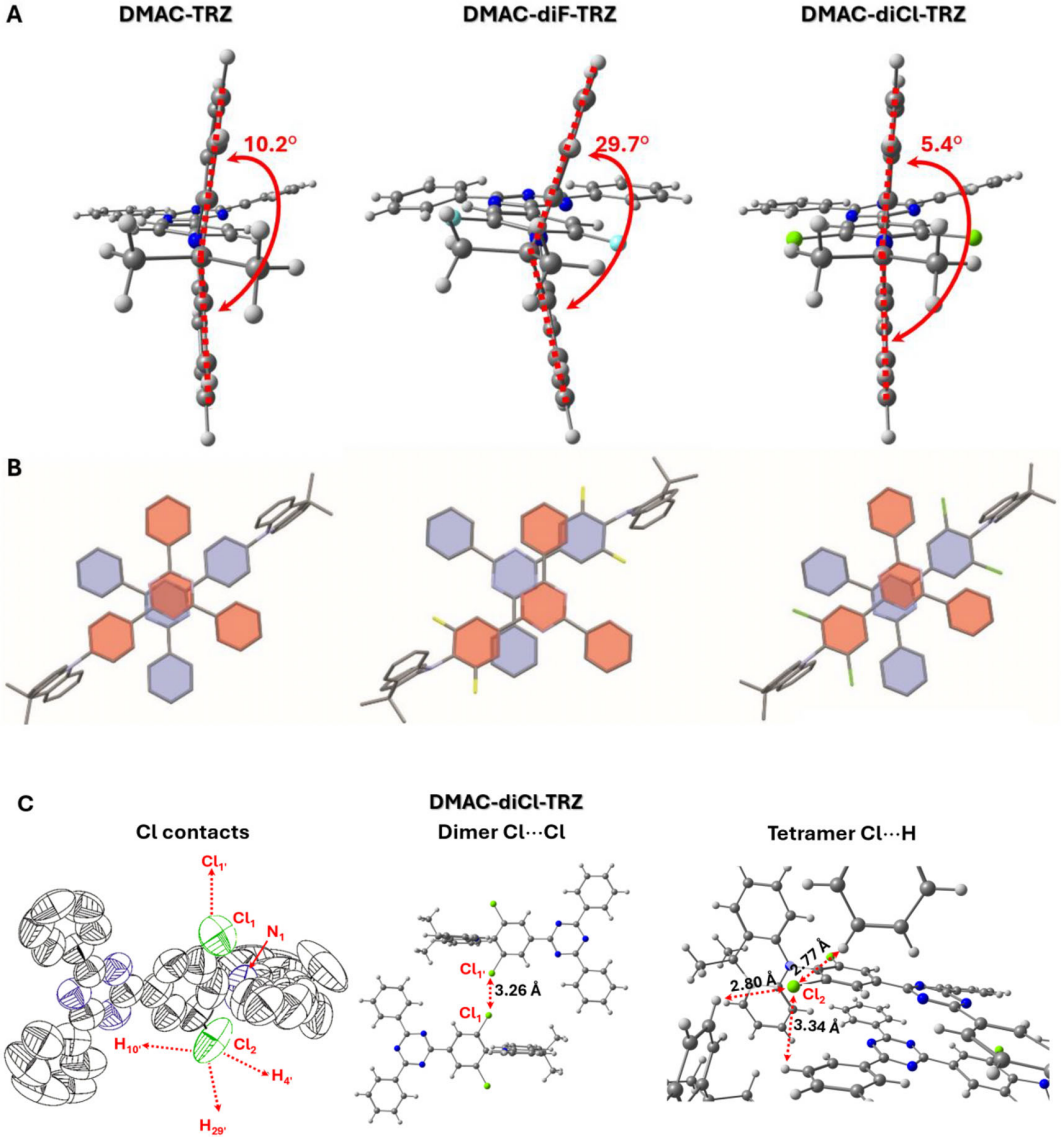
SCXRD analysis: (**A**) Molecular structures of **DMAC‐TRZ, DMAC‐diF‐TRZ**, and **DMAC‐diCl‐TRZ** viewed along the N_1_–C_16_ bond. (**B**) Molecular arrangement in an acceptor–acceptor (A–A) dimer in the crystal. (**C**) Molecular structure with 99% probability ellipsoids and the arrangement of aggregates formed through Cl contacts in the crystal of **DMAC‐diCl‐TRZ**.

#### 2.2.2 Intermolecular Interactions

The crystal structures also differ substantially in the nature of intermolecular contacts that may influence electronic coupling and radiative processes. To identify the most probable sites of exciton localization within the crystal, the intermolecular contacts involving π‐electron density were analyzed.

The most characteristic type of π–π interaction observed in all investigated crystals involves intercalation of the triazine acceptor planes, leading to the formation of dimer, where two TRZ fragments engage in tail‐to‐tail π–π stacking (Figure 3B and Figure S2). In **DMAC‐TRZ** and **DMAC‐diCl‐TRZ**, such an A‐A dimer presents with an interplanar distance of 3.38 Å and a lateral offset of 0.6 and 0.9 Å, respectively, between the triazine ring centroids. In the respective A‐A dimer of **DMAC‐diF‐TRZ**, the interplanar separation decreases to 3.29 Å, but the lateral displacement is increased to 2.5 Å, resulting in much weaker interactions between the π‐systems of the TRZ fragments. Such close stacking can lead to excitonic coupling between the acceptor units, with the strength of this interaction varying among the three compounds due to differences in distance and lateral displacement.

Interestingly, π–π interactions involving another π‐electron‐rich fragment, the DMAC donor, are observed only for **DMAC‐diF‐TRZ** (dimer D–D, Figure S2), where the DMAC units are the least planar. This crystal features a distinct second dimer type, in which two DMAC donor fragments approach each other with a minimum interplanar distance of 3.46 Å, forming an additional potential channel for exciton localization. As a result, the **DMAC‐diF‐TRZ** molecules are aligned in a linear polymeric arrangement through alternating A···A and D···D stacking interactions, represented by the trimer motif D···D–A···A.

Uniquely, in **DMAC‐diCl‐TRZ**, crystal packing is dominated by specific chlorine‐based intermolecular interactions. Each molecule contains two inequivalent Cl atoms (Figure 3C): (i) one engages in a short Cl···Cl contact (3.26 Å) with a neighboring molecule, forming a halogen‐bonded dimer (dimer Cl–Cl), and (ii) the second participates in several Cl···H–C(aromatic) contacts with adjacent donor or acceptor fragments of three neighboring molecules (tetramer Cl–H). As observed previously [20], vibrations of heavy atoms play a critical role in activating SOC. The structural asymmetry and distinct non‐covalent environments of the two Cl atoms are likely to influence both the molecular relaxation pathways and exciton dynamics, potentially restricting the freedom of Cl motion and thereby modulating SOC activation, as discussed in later sections.

### 2.3 Photophysical Properties in the Crystalline Phase

#### 2.3.1 Stationary Photoluminescence (PL)

Although all crystalline emitters maintain high photoluminescence quantum yields (PLQY) reaching 100% for **DMAC‐TRZ** and **DMAC‐diCl‐TRZ**, and 80% for **DMAC‐diF‐TRZ**, halogen substitution causes a significant influence on their photophysical behavior. At room temperature, all three compounds exhibit broad, charge‐transfer (CT) type PL, with a variation in spectral maximum and width (Figure 4A). The emission maximum shifts from 484 nm for **DMAC‐TRZ** to 520 nm for **DMAC‐diF‐TRZ** and then with a reverse blue‐shift to 495 nm in the chlorine derivative. **DMAC‐diCl‐TRZ** also displays the narrowest PL with a full width at half maximum (FWHM) of 0.35 eV compared to 0.40‐0.41 eV for the other two compounds (Table 1). This spectral sharpening correlates with the expected higher structural order and the observed strictly orthogonal D‐A configuration. On the other hand, the pronounced red shift observed for **DMAC‐diF‐TRZ** relative to **DMAC‐diCl‐TRZ** is somewhat non‐intuitive when considering emission from isolated species, since the decreased planarity of the DMAC donor should normally increase the CT energy. Such a discrepancy may indicate the presence of distinct aggregation effects, like D–D dimers or D···D–A···A trimers, that stabilize the ^1^CT‐S_0_ transition in the fluorinated derivative but are absent in the chlorine analog.

**FIGURE 4:**
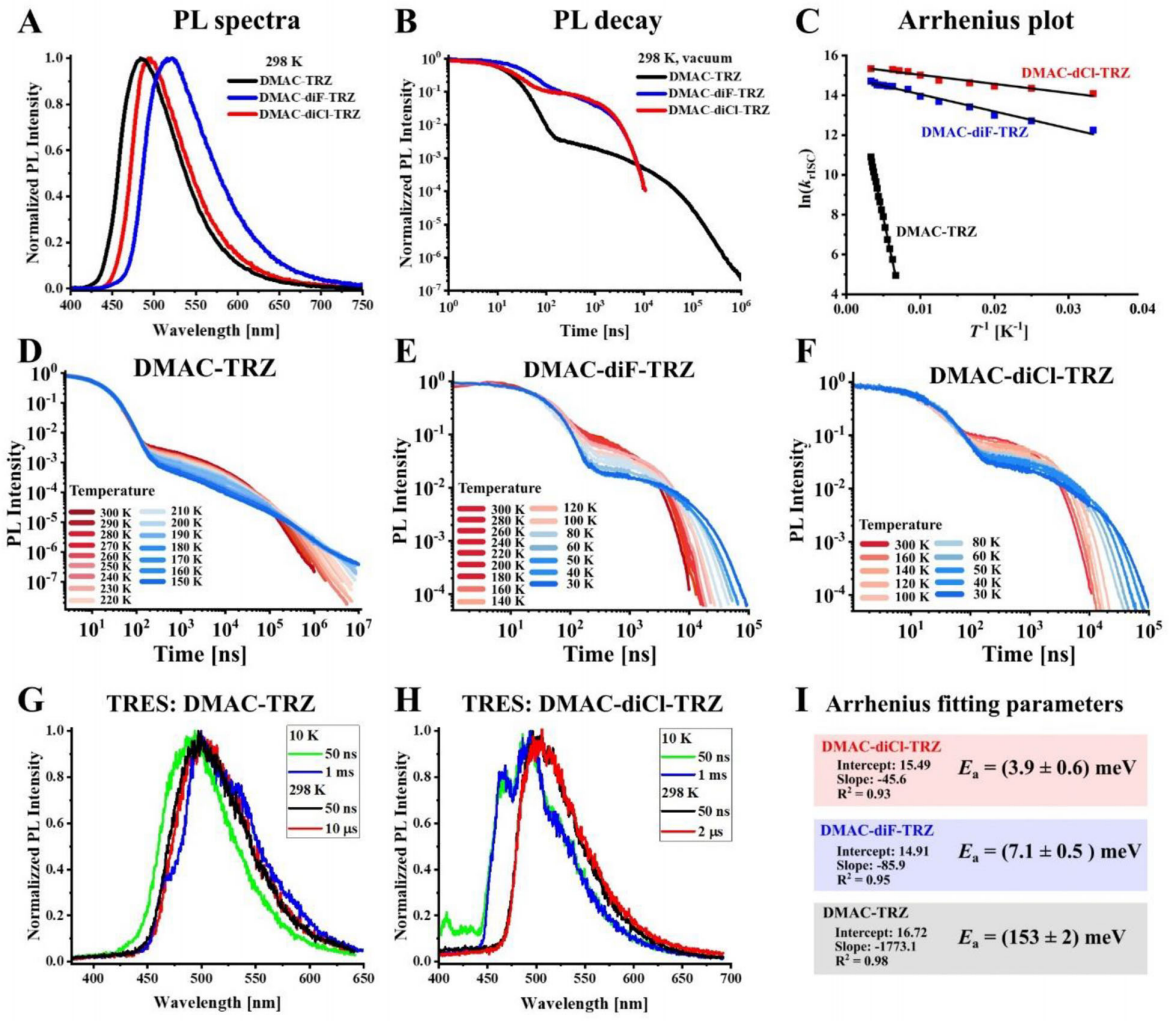
Photoluminescence properties of studied crystals. (A) Steady‐state PL spectra at room temperature. (B) PL decay profiles. (C) Arrhenius plot of ln(*k*_rISC_) versus *T* ^−1^. Photoluminescence decay curves at various temperatures for **DMAC‐TRZ** (D), **DMAC‐diF‐TRZ** (E), and **DMAC‐diCl‐TRZ** (F). Time‐resolved emission spectra (TRES) at various temperatures for **DMAC‐TRZ** (G) and **DMAC‐diCl‐TRZ** (H). (I) Arrhenius fitting parameters.

**TABLE 1:** Photoluminescence and photophysical parameters of the investigated crystals at 298 K.

Compound	PLQY	PL_max_	FWHM	*τ*_PF_	*τ*_DF_	*k*_r_	*k*_ISC_	*k*_rISC_	*E*_a_
	[%]	[nm]	[eV]/[nm]	[ns]	[µs]	[10^7^ s^−1^]			[meV]
**DMAC‐TRZ**	100	484	0.40/82	15.8	42.2	2.7	3.6	5.4 × 10^−3^	153
**DMAC‐diF‐TRZ**	80	520	0.41/90	43.1	2.0	0.47	1.7	0.24	7.1
**DMAC‐diCl‐TRZ**	100	495	0.35/73	20.5	1.2	0.55	4.3	0.65	3.9

#### 2.3.2 PL Dynamics

Time‐resolved PL measurements reveal both prompt fluorescence (PF) and delayed fluorescence (DF) components in all samples (Figure 4B, Table 1). The PF lifetime (*τ*_PF_) shows a non‐monotonic trend across the series. It first increases sharply from 15.8 ns in **DMAC‐TRZ** to 43.1 ns in **DMAC‐diF‐TRZ**, due to a substantial reduction of the radiative rate constant (*k*_r_, from 2.7 × 10^7^ to 5 × 10^6^ s^−1^) and the ISC rate constant (*k*_ISC_, from 3.6 × 10^7^ to 1.7 × 10^7^ s^−1^). Such changes typically indicate the enhanced CT character and/or stronger separation of HOMO and LUMO. In **DMAC‐diCl‐TRZ**, *k*_r_ remains close to 5 × 10^6^ s^−1^, yet its *τ*_PF_ drops again to 20.5 ns due to the *k*_ISC_ increase up to 4.3 × 10^7^ s^−1^ representing an almost threefold increase as compared with the fluorinated analogue. Such behavior indicates strong separation of the frontier molecular orbitals in the halogenated derivatives, with a pronounced Cl‐induced spin‐flip enhancement.

The delayed‐fluorescence lifetime (*τ*_DF_) changes more significantly: it decreases from 42.2 µs (**DMAC‐TRZ**) to 2.0 µs (**DMAC‐diF‐TRZ**) and reaches minimum of 1.2 µs for **DMAC‐diCl‐TRZ**. Correspondingly, the rISC rate constant (*k*_rISC_) increases steeply across the series, reaching 6.5 × 10^6^ s^−1^ for **DMAC‐diCl‐TRZ**, over two orders of magnitude higher than in the parent compound and among the largest reported for light‐atom TADF systems [22].

#### 2.3.3 Temperature‐ and Time‐Resolved PL

Upon cooling, as thermal relaxation is progressively suppressed, the prompt fluorescence spectra of all crystals show a pronounced blue shift (Figures 4G,H and S3). At 10 K, the long‐lived emission of **DMAC‐TRZ** is significantly red‐shifted vs. PF, most likely indicating phosphorescence. None of the halogenated emitters follow this pattern: prompt and delayed emission spectra remain identical even at cryogenic temperatures, evidencing the delayed fluorescence. Notably, over 180 K, both PF and DF spectra become narrower and lose the vibronic structure, suggesting that temperature‐dependent relaxation processes play an important role in the emission mechanism.

Temperature‐dependent PL decays (30–300 K) confirm that DF has a thermally activated nature in all crystalline emitters (Figures 4D–F and Figure S4). For **DMAC‐TRZ**, the delayed component exhibits a strong temperature dependence. Its delayed fluorescence lifetime reaches the millisecond regime below 150 K, where it starts to compete with phosphorescence. The analysis of ln(*k*_rISC_) dependence on T (Tables S2–S4) and Arrhenius plot (Figure 4C) yields rISC activation energy (*E*_a_) of 153 meV, which is consistent with the substantial differences of its fluorescence and phosphorescence spectra (Figure 4G), confirming that the *T*_1_ triplet state has a large contribution of ^3^LE character. In contrast, both halogenated derivatives show markedly weaker temperature dependence, consistent with significantly smaller Δ*E*_ST_ and much faster rISC rates: near 10K, the *τ*_DF_ values reach 30 µs and 15 µs for the fluorine and chlorine derivatives, respectively. The *E*_a_ values of the halogenated crystals decrease sharply reaching 7.1 meV for **DMAC‐diF‐TRZ** and an exceptionally low value of 3.9 meV for **DMAC‐diCl‐TRZ** (Figure 4I). Such nearly degenerate S_1_ and T_1_ states evidence their strong CT character and reflect several key factors: (*i*) the targeted ortho effect of the halogen substituents, and (*ii*) the specific molecular alignment within the crystal lattice, namely D···D π–π interactions in the fluorinated derivative and a highly orthogonal D‐A arrangement in the chlorinated derivative. Such low *E*_a_ values explain why, as mentioned above, TADF is observed at cryogenic temperature and why any phosphorescence, if present, cannot be spectrally resolved from fluorescence. Taken together, these observations confirm that the applied molecular design achieves near‐degeneracy of the *S*_1_ and *T*_1_ states, exactly as intended.

#### 2.3.4 Spin‐Orbit Coupling

Equally important is the intercept of the Arrhenius plot (ln *A*), where the preexponential factor A is proportional to SOC^2^ [20]. The intercept is noticeably higher for the chlorinated derivative (Figure 4I), corresponding to an estimated increase of SOC by a factor of approximately 1.35 in **DMAC‐diCl‐TRZ** relative to **DMAC‐diF‐TRZ**. This provides direct evidence of a moderate heavy‐atom effect of chlorine on spin‐flip transitions.

### 2.4 Radioluminescence and Scintillation Performance

#### 2.4.1 Radioluminescence at Ambient Conditions

Because the PL experiments indicate superior TADF properties of the halogenated derivatives, particularly the chlorine‐substituted one, we further performed radioluminescence measurements. We first recorded the pulse‐height spectra at room temperature for **DMAC‐diF‐TRZ** and **DMAC‐diCl‐TRZ**, providing an estimate of the scintillation light yield (Figure 5A). Numerous unsuccessful attempts to obtain macroscopic **DMAC‐TRZ** crystals prevented reliable pulse‐height measurements for the parent compound. In contrast, **DMAC‐diCl‐TRZ** reproducibly formed sufficiently large, optically homogeneous crystals suitable for quantitative gamma‐ray measurements, highlighting an additional practical advantage of chlorine substitution. Under 59.5 keV gamma excitation from a ^241^Am source the spectra of both materials exhibit clear full‐energy peaks (FEP), which—after necessary calculations—provide the light yield values of 12 300 photons/MeV for **DMAC‐diF‐TRZ** and 22 900 photons/MeV for **DMAC‐diCl‐TRZ** (Table S5). These values should not, however, be directly compared with literature data, as these are most often provided for excitation at 662 keV. Most scintillators exhibit a certain degree of nonproportionality of the light yield with respect to excitation energy, typically showing an enhancement with increasing energy. On the other hand, a common feature of many organic and low‐Z compounds [23] is the absence of a clearly resolved FEP under such excitation conditions, which complicates or even prevents a reliable determination of light yield. We attempted some measurements using a ^137^Cs source, and only in the case of **DMAC‐diCl‐TRZ** did we observe distinct features that allowed an approximate identification of the FEP position and, in turn, an estimation of the light yield. The obtained value is approximately 26 000 ph/MeV (Figure S5A), reflecting the expected nonproportionality behavior. This value can be meaningfully compared with literature data, revealing, for example, a more than threefold higher yield than that of the widely used inorganic BGO scintillator [24], which can be considered promising for many applications.

**FIGURE 5:**
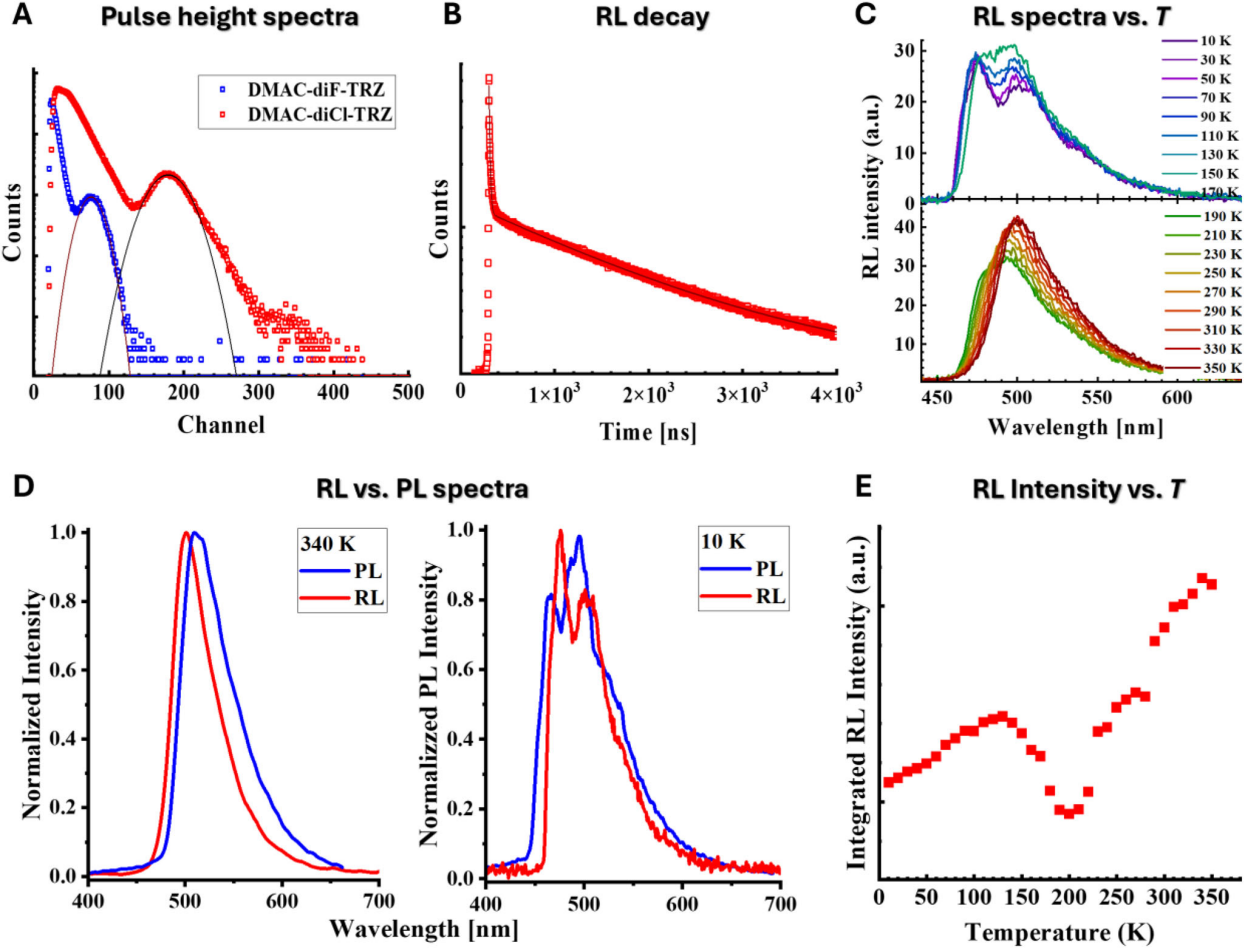
(A) ^241^Am pulse‐height spectra of **DMAC‐diF‐TRZ** and **DMAC‐diCl‐TRZ** crystals. (B‐E) Radioluminescence and scintillation characteristics of **DMAC‐diCl‐TRZ**: (B) scintillation decay profile, (C) temperature‐dependent RL spectra, (D) comparison of PL and RL spectra, and (E) RL intensity versus temperature.

As the most promising scintillator candidate, **DMAC‐diCl‐TRZ** was further investigated under pulsed x‐ray excitation. The room‐temperature scintillation decay profile of **DMAC‐diCl‐TRZ**, obtained after deconvolution of the instrumental response using the method described in [25], is presented in Figure 5B. Compared with PL, the scintillation time profile reveals a different interplay between prompt and delayed emissions. Optical excitation directly creates singlet excited states, from which triplets are subsequently populated through ISC. Ionizing radiation, in contrast, first produces ions and secondary electrons, followed by carrier thermalization and recombination, generating a high fraction of triplet excited states [1]. Accordingly, DF dominates the scintillation response of **DMAC‐diCl‐TRZ**, contributing 98% of the total emission with *τ*_DF_ = 1.8 µs, whereas PF accounts for only 2%, with τ_PF_ = 10.8 ns.

The emission decay parameters obtained under PL and scintillation conditions should not, however, be interpreted as identical kinetic quantities. The PL decay primarily reflects the evolution of optically generated molecular excited states, whereas the scintillation time profile additionally contains the kinetics of carrier thermalization, recombination, and population of the emissive states. The pronounced dominance of triplet‐assisted emission reflects not only the direct population of the triplet manifold but also its continued population throughout the ionization‐recombination cascade [26]. This supports the key challenge for modern fast organic scintillators: triplet states must be converted efficiently, but also rapidly, into emissive singlet states.

The more than twofold higher pulse height observed for **DMAC‐diCl‐TRZ** is particularly intriguing, especially considering that the crystal density of fluorinated analogue is higher: 1.244 and 1.356 g/cm^3^, respectively. The enhanced response is therefore most likely attributed to a more efficient conversion of deposited energy into light emission, enabled by faster rISC and shortened DF lifetime. In fact, the light yield increase correlates with the *τ*_DF_ reduction from PL measurements, which is 1.7 times shorter for the chlorinated derivative (Table 1). Therefore, the presence of Cl atoms makes the light emission processes in scintillation more competitive with other deactivation pathways.

#### 2.4.2 Temperature‐Dependent Radioluminescence and Photoluminescence

We proceed with the analysis of the spectral RL properties using temperature‐dependent spectroscopy. X‐ray excited emission spectra of the **DMAC‐diCl‐TRZ** single crystal recorded between 10 and 350 K are shown in Figure 5C and Figure S5B. At 10 K, the RL spectrum exhibits a maximum at 475 nm and pronounced vibronic structure. The latter gradually disappears upon heating and is no longer resolved above 200 K, while RL_max_ shifts to 490 nm. With the further temperature increase, the RL band undergoes a moderate red shift to 500 nm, while maintaining a similarly narrow spectral shape. Notably, due to its TADF nature and high structural rigidity, the RL intensity of **DMAC‐diCl‐TRZ** increases with temperature, albeit non‐monotonically (Figure 5E), in contrast to typical organic fluorescent scintillators that exhibit strong thermal quenching already above several tens of Kelvin [27].

A comparison of RL and PL temperature dependences points on important similarities and differences (Figure 5D). At cryogenic temperatures, both luminescence types yield broad, blue‐shifted emission dominated by fluorescence/TADF without detectable phosphorescence contribution. Importantly, the emission centroid of the RL spectrum closely matches the PL component at 10 K, indicating that RL has a fluorescent/TADF nature. However, the vibronic structures observed in RL and PL at 10 K are not identical, suggesting that photoexcitation and ionizing‐radiation excitation access different vibrational subsets of emissive configurations within the crystal. At higher temperatures (210–350 K) as exciton relaxation becomes more efficient, both PL and RL spectra become narrower and red‐shifted. Such a change in the emission profile may indicate the involvement of different emissive species, possibly associated with the formation of dimeric aggregates (Figure 3B). The transition between these distinct emissive species is also reflected in the temperature dependence of the RL intensity, which shows a pronounced minimum around 200 K (Figure 5E). Moreover, at high temperatures the RL spectrum remains approximately 15 nm blue‐shifted relative to PL. Together with the altered scintillation kinetics, this spectral shift supports the presence of a less fully relaxed, non‐equilibrium excited‐state population under ionizing excitation. In fact, it was suggested previously that high‐energy excitation can access upper excited‐state manifolds and relaxation pathways that are not populated under direct optical excitation [28]. Thus, under RL conditions, emission may originate from both relaxed and higher‐energy excitonic states before complete equilibration.

To reveal the possible role of charge trapping in the mechanism of RL, low‐temperature thermoluminescence measurements were performed in a 10–350 K temperature range (Figure S5C). The ltTL glow curve is structurally very simple, exhibiting only a single relatively broad peak centered at 70–80 K. However, its intensity is so low that the contribution of traps to the energy transfer from the crystal lattice to the luminescence centers is estimated to be less than 1%. Therefore, **DMAC‐diCl‐TRZ** can be classified as a “trap‐free material,” and its superior scintillating properties under the increase of temperature are explained by enhanced thermally activated rISC.

#### 2.4.3 Operational Stability and X‐Ray Imaging

Having established the high scintillation LY and the dominant role of triplet‐assisted emission, we next examined properties relevant to practical application: operational stability and x‐ray imaging capability.

To evaluate short‐term operational reproducibility, the RL intensity of **DMAC‐diCl‐TRZ** was continuously monitored during repeated x‐ray irradiation while varying the tube voltage from 15 to 45 kV and the current from 6 to 10 mA (Figure S6). For each irradiation condition, the signal rapidly reached a stable plateau, with no systematic decrease during exposure or cumulative loss over the entire measurement sequence. The RL intensity increased reproducibly with increasing tube voltage and current and returned to the background level after each irradiation period. Thus, **DMAC‐diCl‐TRZ** shows a reversible and reproducible response to repeated changes in x‐ray excitation conditions, with no detectable bleaching under the conditions investigated, demonstrating excellent short‐term operational stability under repeated x‐ray exposure.

Encouraged by this stability, we next evaluated whether the strong RL response of **DMAC‐diCl‐TRZ** could be directly translated into x‐ray imaging. A simple polycrystalline scintillation screen was prepared by mechanically distributing **DMAC‐diCl‐TRZ** ground powder over a transparent substrate. A small microchip, approximately 3 mm in width, was placed between the x‐ray source and the scintillation screen (Figure 6A–E). Under x‐ray irradiation, the transmitted x‐ray pattern was converted by the screen into visible emission and recorded with a digital camera. Despite the simplicity of the screen preparation and imaging setup, the resulting image clearly resolves the outline of the microchip and its individual pins with widths below 0.5 mm. The corresponding intensity profile confirms periodic spatial contrast associated with the pin structure (Figure 6E).

**FIGURE 6:**
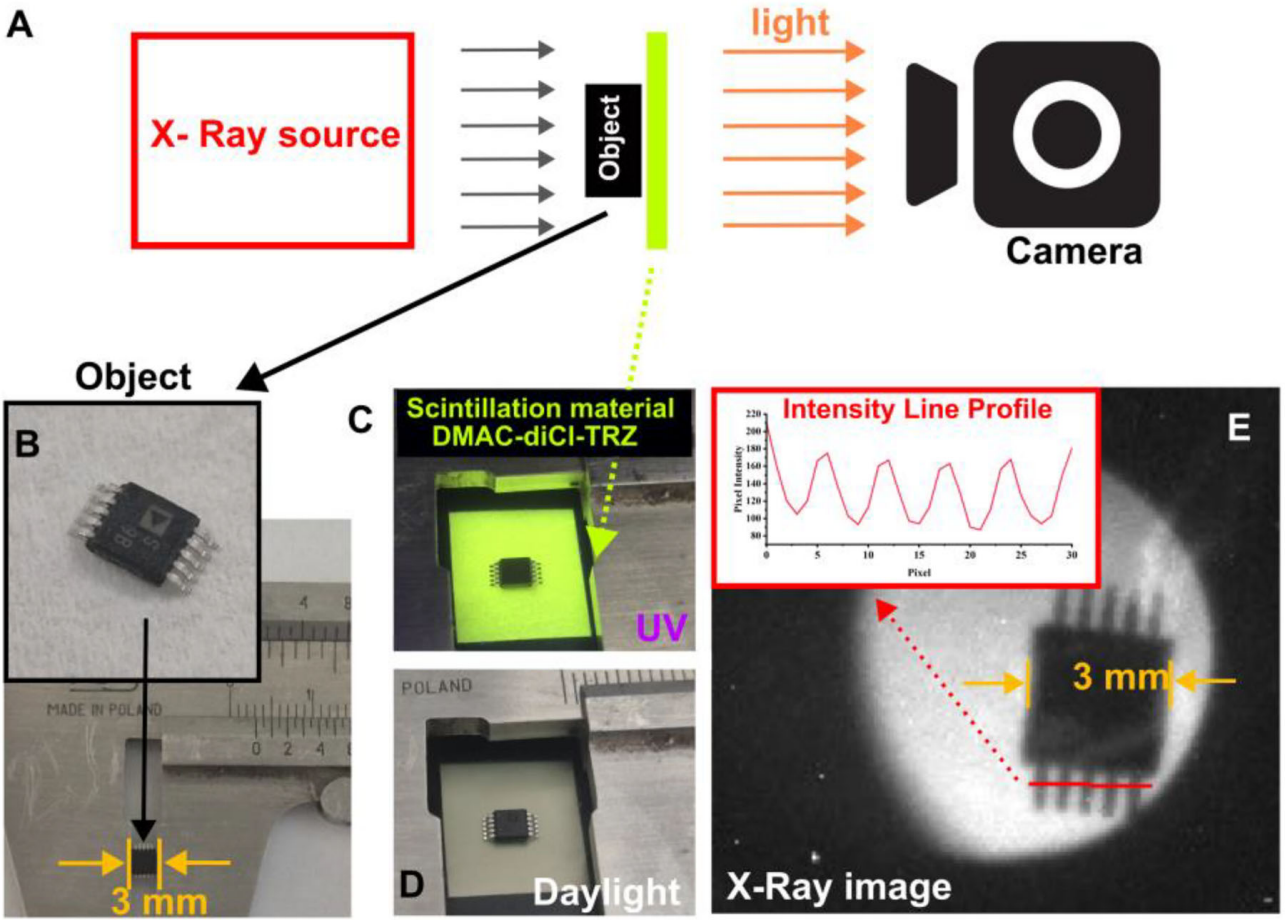
Proof‐of‐concept x‐ray imaging using a **DMAC‐diCl‐TRZ** scintillation screen. (A) Schematic illustration of the experimental setup. (B) A 3 mm‐wide microchip used as the test object. (C, D) Polycrystalline **DMAC‐diCl‐TRZ** scintillation screen under UV excitation and daylight, respectively. (E) X‐ray image of the microchip recorded at a tube voltage of 45 kV and a current of 10 mA; the inset shows the intensity line profile across several microchip pins.

It is important to emphasize that given the efficient radioluminescence response of the material, the spatial resolution achieved in test is predominantly limited by morphological and technological factors, such as the quality of the polycrystalline sample distribution process on the screen rather than the intrinsic photophysical performance of the **DMAC‐diCl‐TRZ** emitter itself. We believe that these results successfully validate the bright radioluminescence and the promising potential of **DMAC‐diCl‐TRZ** for future imaging screens, where optimized, industrial fabrication methods could fully exploit its capabilities.

### 2.5 DFT Calculations

To gain deeper insight into the observed photophysical behavior, we performed DFT and TD‐DFT calculations for the S_1_‐T_n_ manifold of selected monomeric and aggregated species (dimers, trimers, and tetramers) observed in the crystal structures.

#### 2.5.1 Energetics of Aggregated Species

Within the monomeric species, the calculated S_1_ energies decrease in the order **DMAC‐diF‐TRZ > DMAC‐TRZ > DMAC‐diCl‐TRZ** (Figure S8). This trend contradicts the experimentally observed emission energies, indicating that in at least some of these crystals the monomers are not the primary sites responsible for radiative exciton deactivation. As for the aggregated species, formation of the A‐A dimer increases the S_1_ energy by 60–100 meV (Table S6), demonstrating that π–π stacking of triaryl‐*s*‐triazine leads to the destabilization of the CT state. Calculations suggest that **DMAC‐diF‐TRZ** owes its lowest luminescence energy to the donor‐donor π‐π interactions. The representative D‐D dimer and the extended D···D‐A···A trimer show markedly reduced S_1_ energies: according to M06‐2X, the S_1_ state is stabilized by 76 meV in the D‐D dimer and by 127 meV in the D···D‐A···A trimer (Figure 7B). As for the decrease in *E*_a_ observed in experiment, this trend is also supported by the predicted aggregate properties: M06‐2X reproduces the very small ^1^CT–^3^CT gaps (Table S6), whereas CAM‐B3LYP, although overestimating their absolute values, predicts a large reduction from 235 meV in the **DMAC‐TRZ** dimer to 91 meV in the D···D‐A···A trimer of **DMAC‐diF‐TRZ** (see Table S7 and corresponding discussion in Supporting Information).

**FIGURE 7:**
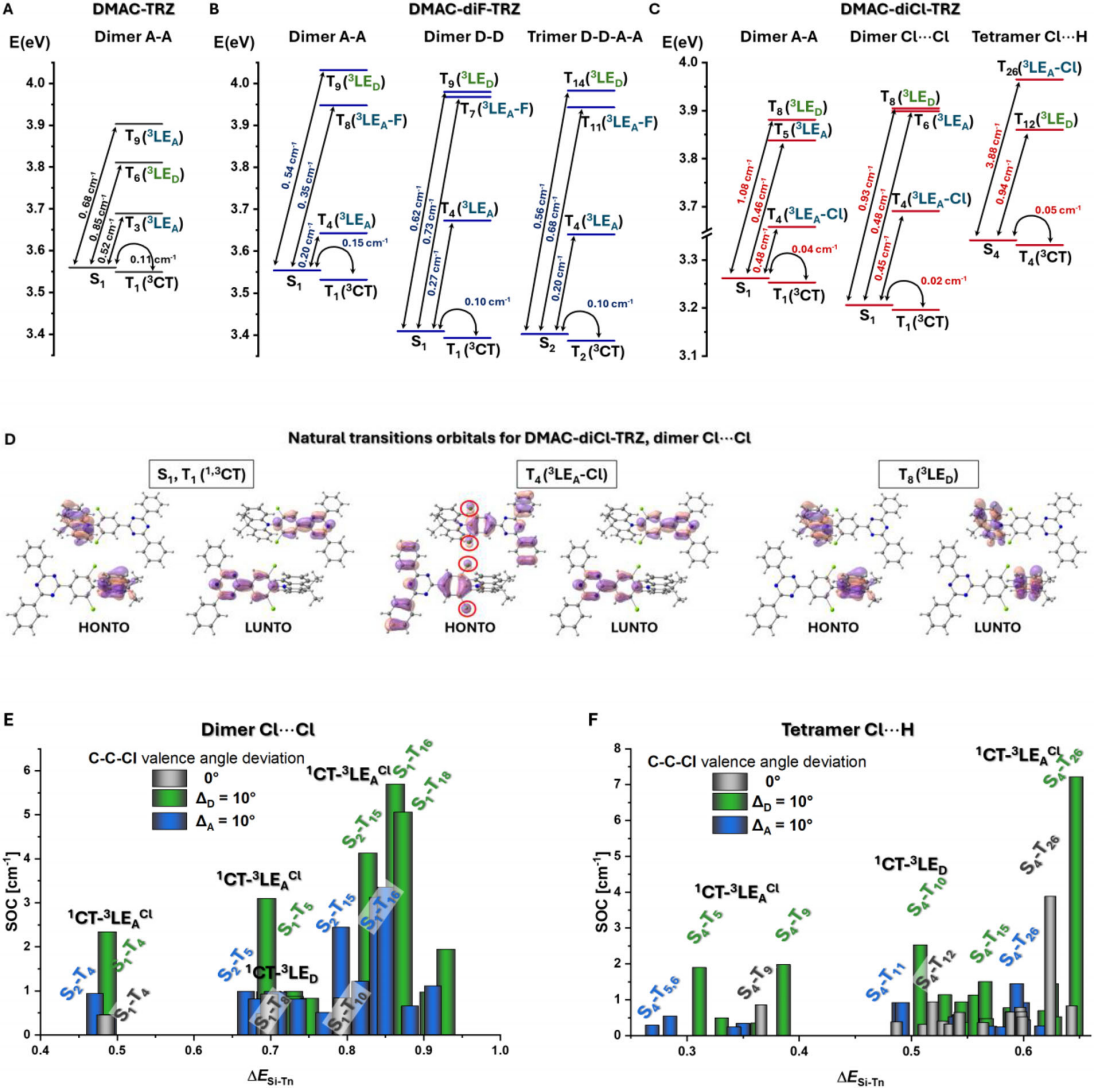
Summary of computational analysis performed at the M06‐2X/def2‐SVP level of theory. (A–C) Energy alignment of excited states with significant SOC for the investigated molecular clusters. (D) Natural transition orbitals for the Cl···Cl dimer. (E, F) SOC values for selected S‐T transitions with SOC > 0.3 cm^−1^ as a function of the singlet‐triplet energy gap for the Cl···Cl dimer (E) and Cl···H tetramer (F) of **DMAC‐diCl‐TRZ**, calculated upon variation of the C–C–Cl valence angle. Δ_A_ and Δ_D_ indicate changes in the direction of the acceptor and donor, respectively. For the tetramer, only transitions involving *S*_4_ state were analyzed, localized on the monomer unit in which the Cl geometry was perturbed. Complete transition parameters are provided in Table S8.

As **DMAC‐diCl‐TRZ** crystal is unique in possessing two inequivalent chlorine atoms, we also analyzed dimeric and tetrameric species involving the characteristic Cl···Cl and Cl···H contacts (Figure 3C and Table S6). The Cl···Cl dimer changes the S_1_ energy by only about 40 meV, whereas the Cl···H tetramer destabilizes the locally excited singlet state by more than 170 meV. Thus, the local environment of the Cl derivative destabilizes the CT state and contributes to its blue‐shifted emission relative to **DMAC‐diF‐TRZ**.

#### 2.5.2 Spin‐Flip Pathways and Vibrational Modulation of SOC

Before discussing spin‐flip channels, we note that among tested approaches no single functional simultaneously reproduces the very small CT–CT gaps and a realistic ordering of the higher LE states. M06‐2X better describes the low‐energy CT–CT separations, whereas CAM‐B3LYP places several higher triplet states closer to the relevant singlets (see discussion in Supporting Information). We therefore treat the predicted LE‐CT energy gaps with caution and mainly identify the groups of LE states that couple with singlet states and how vibrations influence these couplings.

The energy alignment and SOC between *S*_1_ and *T*_n_ states was analyzed, starting from the S_1_–T_1_ transition. In **DMAC‐diF‐TRZ**, the energy gap *S*_1_–*T*_1_ is the most sensitive to the D···D interactions: Δ*E*_ST_ changes from 22 meV in the A‐A dimer to 16 meV in the D···D‐A···A trimer. Calculations support the above‐mentioned experimental conclusions: S_1_ and T_1_ states have strong CT character involving either LUMO devocalized on the extended A···A acceptor or HOMO on the D···D fragment, respectively (Figure S9). In **DMAC‐diCl‐TRZ**, Δ*E*_ST_ decrease to 9–10 meV, which correlates well with the experimental observations. It is almost insensitive to intermolecular interactions, supporting that the orthogonal D‐A arrangement is the most crucial factor for achieving the S_1_–T_1_ degeneracy.

Due to strong CT character, ⟨S_1_∣Ĥ_SO_∣T_1_⟩ correlates with the aforementioned degree of orthogonality within the D–A framework, which regulates D–A conjugation and thus changes in orbital momentum (Δ*L*) of singlet‐triplet transition. Among the A‐A dimers, ⟨S_1_∣Ĥ_SO_∣T_1_⟩ decreases from 0.15 cm^−1^ in **DMAC‐diF‐TRZ** with the most bent DMAC and 0.11 cm^−1^ in **DMAC‐TRZ** down to 0.02 cm^−1^ in **DMAC‐diCl‐TRZ** with the most planar DMAC and orthogonal D‐A alignment (Figure 7A–C). The intermolecular Cl···Cl and Cl···H contacts present in **DMAC‐diCl‐TRZ** do not significantly increase ⟨S_1_∣Ĥ_SO_∣T_1_⟩ (Figure 7C), indicating the lack of heavy‐atom effect on the almost forbidden *S*_1_–*T*_1_ transition. In general, the discussed trend, however, differs from the experimental observations: low values of calculated ⟨S_1_∣Ĥ_SO_∣T_1_⟩ indicate that only in **DMAC‐diF‐TRZ**, the *T*_1_–*S*_1_ channel can contribute to rISC. Especially in **DMAC‐diCl‐TRZ**, rISC mechanism should extend far beyond the direct ^1^CT–^3^CT transition.

We thus further examined the higher‐lying triplet manifold, which provides stronger coupling with S_1_. In almost all studied cases, higher excited states with significant SOC are localized on the acceptor (^3^LE_A_), with ⟨^1^CT∣Ĥ_SO_∣^3^LE_A_⟩ reaching representative values of 0.52, 0.20, and 0.37 cm^−1^ for **DMAC‐TRZ** (S_1_‐T_3_), **DMAC‐diF‐TRZ** (S_1_‐T_4_), and **DMAC‐diCl‐TRZ** (S_1_‐T_4_), respectively. Another group of locally excited states, the ^3^LE_D_ manifold, is dominated by electronic density on the donor fragment (Figure S9). Despite the insignificant contribution of electronic density from halogen atoms, the vicinity of F and Cl significantly modulates ⟨^1^CT∣Ĥ_SO_∣^3^LE_D_⟩ from 0.85 cm^−1^ (T_6_) in **DMAC‐TRZ**, to 0.54 cm^−1^ (T_9_) in **DMAC‐diF‐TRZ**, and 1.08 cm^−1^ (T_8_) in **DMAC‐diCl‐TRZ**. The trends in SOC predicted for the ^3^LE_A_‐^1^CT and ^3^LE_D_‐^1^CT transitions correlate well with the experimental observations, indicating a significant involvement of ^3^LE states in the rISC mechanism of the studied crystals.

It is particularly noteworthy that two chemically similar halogens cause such different effects on both ^3^LE_D_‐^1^CT and ^3^LE_A_‐^1^CT transitions. The strong electron‐withdrawing character of fluorine appears to suppress SOC for transitions involving a large change in Δ*L*, whereas chlorine, likely due to its more polarizable valence shell, does not reduce the ⟨^1^CT​∣Ĥ_SO_​∣^3^LE_A_​⟩ to the same extent and even enhances ⟨^1^CT∣Ĥ_SO_∣^3^LE_D_⟩. This effect is further illustrated in the Cl···H tetramer (Figure 7C), where H‐bond perturbation of the chlorine electron density increases ⟨^1^CT∣Ĥ_SO_∣^3^LE_A_⟩ to 3.8 cm^−1^, over six times higher than in the fluorinated analogue. Such sensitivity to subtle electronic‐density changes motivated us to take a closer look at intermolecular Cl‐based contacts and analyze how vibrational motions within the crystal environment can modulate SOC.

The anisotropic displacement ellipsoids in the crystal structure indicate that the chlorine atoms can undergo significant positional fluctuations (Figure 3C). In particular, Cl_2_ predominantly oscillates through changes in the ∠C–C–Cl valence angle, moving alternately closer to either the donor or the acceptor, whereas Cl_1_ exhibits similar motions but with additional deviations out of the linker aryl plane. Therefore, the ∠C–C–Cl valence angle was varied within the maximum range of ±10°, as estimated from the displacement ellipsoids. To account for the two inequivalent chlorine atoms within the molecule, the calculations were performed separately for both local environments: (i) the Cl_1_···Cl_1’_ dimeric motif, where the motion of one or both neighboring chlorine atoms can influence not only the local thermodynamics but also the electronic charge distribution on each Cl atom, and (ii) the Cl_2_···H tetrameric arrangement, where interactions between chlorine and adjacent C‐H or aromatic fragments can either orient or hinder such vibrational motions. Thermodynamics of such vibrations is summarized in Table S9 and discussed in detail in SI.

For clarity, the distortion toward the acceptor is denoted as Δ_A_, while that toward the donor is denoted as Δ_D_. In the Cl···Cl dimer, motions of Cl_1_ strongly enhance SOC between ^1^CT and ^3^LE states (Figure 7E). For example, for the lowest energy ^3^LE_A_ state (*T*_4_), the ⟨^1^CT∣Ĥ_SO_∣^3^LE_A_⟩ value increases from 0.45 cm^−1^ (equilibrium) to 0.94 cm^−1^ (Δ_A_) and up to 2.3 cm^−1^ (Δ_D_). For selected higher‐energy spin‐flip channels SOC reaches 5.7 cm^−1^, ⟨S_1_∣Ĥ_SO_∣T_16_⟩, which may contribute under high‐energy excitation conditions such as scintillation. In the Cl···H tetramer, the relevant singlet state, *S*_4_, localized on the perturbed molecule (see Figure S9), participates in several low‐gap S–T transitions whose SOC values are strongly modulated by chlorine motion. For the Δ_A_ distortion, the SOC for the lowest‐energy channels (S_4_–T_6_, S_4_–T_11_) increases moderately to 0.55–0.92 cm^−1^, while Δ_D_ distortions produce much stronger enhancement, reaching 1.9 cm^−1^ for S_4_–T_5_ and peaking at 7.2 cm^−1^ for S_4_–T_26_ (Figure 7F). Overall, the Cl motion toward the acceptor activates a number of moderately coupled transitions, whereas motion toward the donor produces the strongest SOC enhancement, providing the most efficient spin‐flip channels.

We thus conclude that despite the rigidity of the crystalline environment and the presence of specific intermolecular contacts, chlorine vibrations play an important role in the TADF mechanism in crystalline **DMAC‐diCl‐TRZ**. For photoluminescence, where the excitation energies are relatively low and the emissive manifold is dominated by T_1_, the enhancement of the ^1^CT‐^3^LE_A_ transition with its comparatively small energy gap is of primary importance for accelerating rISC. The contribution of higher‐lying triplet states also with enhanced SOC with S_1_ becomes particularly relevant under radioluminescence conditions, where high‐energy excitation increases the probability of populating upper triplet levels. In such situations, strong coupling between these high‐lying triplets and singlet states can facilitate rapid spin‐flip processes [28], effectively enhancing the mixing of electronic state multiplicities and opening additional rISC channels that are not accessible under standard optical excitation.

#### 2.5.3 Correlation Between Theory and Experiment

The combined experimental and theoretical analysis clarifies the mechanism of TADF in the investigated systems. In general, TADF arises from thermally activated rISC between singlet and triplet states, where the efficiency of spin flipping is determined by the interplay between the Δ*E*_ST_ and SOC. In the parent **DMAC‐TRZ**, rISC occurs mainly through the direct T_1_‐S_1_ transition, enabled by the different nature of the two states and the moderate SOC between them. However, the lowest triplet state retains a significant locally excited ^3^LE_A_ character, resulting in a comparatively large activation energy and a long delayed‐fluorescence lifetime. Despite the lack of suitable crystals for RL tests and direct comparison with photophysical parameters, previous works reported this material as a promising scintillator [1].

In the halogenated derivatives the situation changes due to stabilization of charge‐transfer states due to intermolecular interactions and/or nearly orthogonal D‐A alignment in the crystal. In **DMAC‐diF‐TRZ**, this stabilization produces an exceptionally small activation barrier for rISC, although the strongly electron‐withdrawing fluorine atoms simultaneously reduce SOC with locally excited states. As a result, the TADF mechanism remains largely governed by the T_1_‐S_1_ channel while intermolecular D···D interactions mainly modify the emission energy and Δ*E*_ST_.

The most efficient emitter and scintillator, **DMAC‐diCl‐TRZ**, follows more advanced mechanism. While *E*_a_ becomes even smaller, the CT‐type T_1_ state can couple with several higher‐lying triplet states of LE character. The presence of chlorine atoms, and in particular their motion within the intermolecular space permitted by the crystal packing, dynamically enhances SOC through multiple channels. Therefore, rISC proceeds through vibronically assisted transitions within a near‐degenerate T_1_‐S_1_ manifold, explaining the low activation energy, efficient triplet harvesting, and strong radioluminescence observed.

Calculations also help to understand the temperature‐dependent spectral changes in PL and RL. At low temperatures the blue‐shifted emission originates from the higher energy π–π stacked A···A dimers stabilized by crystal packing. The increasing temperature enables thermal relaxation which disrupts A···A interactions resulting in the bathochromic shift of PL and RL spectra originating from monomers or different aggregate species.

## 3 Conclusion

In summary, we demonstrate that the efficiency of crystalline TADF scintillators can be controlled not only through electronic structure engineering but also through targeted vibrational dynamics of heavy atoms embedded in the lattice. Precise single‐atom molecular engineering shows that positioning halogens in the ortho position to the donor in a donor‐acceptor system significantly enhances triplet‐mediated light emission, including radioluminescence. A comparative investigation of **DMAC‐TRZ** derivatives shows that halogen substitution strongly reduces the S_1_‐T_1_ energy gap, enabling a record‐low rISC activation energy of 4 meV and efficient TADF even at cryogenic temperatures. Additionally, chlorine introduces vibrational enhancement of spin‐orbit coupling. Structural analysis reveals various intermolecular interactions that preserve significant positional flexibility of chlorine atoms in the crystal. Theoretical calculations show that chlorine motion strongly enhances spin‐orbit coupling between charge‐transfer singlet states and locally excited triplet states. Consequently, reverse intersystem crossing in a **DMAC‐diCl‐TRZ** scintillator can proceed through a network of vibronically assisted spin‐flip channels involving higher triplet states rather than a single T_1_‐S_1_ transition. This mechanism explains the combination of an ultralow activation barrier, rapid microsecond delayed fluorescence, and efficient radioluminescence observed experimentally. The chlorinated crystal exhibits an estimated light yield of 26 000 photons MeV^−1^ and represents one of the most efficient organic crystalline scintillators reported to date [29], owing to submicrosecond rISC. More broadly, these results indicate that dynamic spin–vibronic coupling mediated by medium‐heavy atoms is a powerful approach in the molecular design for next‐generation organic scintillators and TADF materials. The presented results show also that molecular clustering in the crystal can efficiently modulate both photo‐ and radioluminescent performance without introducing significant non‐radiative losses. The intermolecular interactions stabilize the lattice while preserving the chlorine vibrational dynamics that facilitate spin‐orbit coupling.

From the perspective of engineering of crystal‐phase TADF scintillators, the structural consequences of F and Cl substitution are fundamentally different. **DMAC‐diF‐TRZ** contains the most strongly bent donor and uniquely forms D–D aggregates, which stabilize the S_1_ state and cause the red‐shifted emission, but simultaneously weaken LE‐CT spin‐orbit coupling, resulting in only moderately high scintillation LY. In contrast, **DMAC‐diCl‐TRZ** owes its remarkable triplet‐harvesting efficiency and scintillation performance to the combination of a rigid, planar donor and asymmetric intermolecular contacts that modulate chlorine motion within the crystal. We suggest that these results demonstrate that, in solid‐state TADF scintillators, suppression of structural heterogeneity to achieve a small rISC barrier is most effective when specific SOC channels are additionally activated, either statically or dynamically, as in **DMAC‐diCl‐TRZ**.

## Supporting Information

The Supporting Information includes detailed procedures for the determination of photophysical parameters, additional data on computational calculations, photoluminescence measurements, and NMR spectra. Crystallographic data for the structures reported in this paper have been deposited with the Cambridge Crystallographic Data Centre (CCDC) as supplementary publication numbers CCDC 2543421 and 2543422. These data can be obtained free of charge from The Cambridge Crystallographic Data Centre via www.ccdc.cam.ac.uk/data_request/cif, or by emailing deposit@ccdc.cam.ac.uk, or by contacting The Cambridge Crystallographic Data Centre, 12 Union Road, Cambridge CB2 1EZ, UK; fax: +44 1223 336003.

## Funding

The authors have nothing to report.

## Conflicts of Interest

The authors declare no conflicts of interest.

## Supporting information




**Supporting File 1**: adma74520‐sup‐0001‐SuppMat.docx.

**Supporting File 2**: adma74520‐sup‐0002‐SuppVideos.zip.

## Data Availability

The data that support the findings of this study are available from the corresponding author upon reasonable request.
