# Supplementary material for: Specific Position of Halogen in Crystalline TADF Scintillators Enables Efficient Triplet Harvesting Through Vibrational Modulation of Spin–Orbit Coupling
Source: Adv Mater. 2026 Aug 6;38(56):e74520. doi: 10.1002/adma.74520 (PMC13648889; doi:10.1002/adma.74520)
Supplement: Supplementary file 1 [file ADMA-38-e74520-s001.docx]

**SUPPORTING INFORMATION**

**Specific Position of Halogen in Crystalline TADF Scintillators Enables Efficient Triplet Harvesting through Vibrational Modulation of Spin–Orbit Coupling**

Illia E. Serdiuk,^1^* Michał Mońka,^1^ Artur Sikorski,^3^ Konrad J. Drozdowski,^2^ Mohanad S. Eid,^2^ Krzysztof Wiśniewski,^2^ Damian Trzybiński,^4^ Marcin E. Witkowski,^2^ Winicjusz Drozdowski^2^*

**Contents**

Experimental and computational methods

Single-crystal XRD analysis

Procedure for determination of activation energy

Additional photoluminescence and radioluminescence data

Additional calculation results

NMR spectra

**Experimental and Computational Methods**

**Materials and characterization.** Reagents and solvents for synthesis were of corresponding grades and were used as supplied. Purification was performed by column chromatography using silica gel (70-230 mesh). The ^1^H and ^13^C NMR spectra were recorded on a Bruker AVANCE III 500 instrument (500.13/125.76 MHz) with CDCl_3_ as the solvent; chemical shifts were recorded relative to tetramethylsilane as an internal reference. MALDI-TOF analyses were performed on a Bruker autoflex maX mass spectrometer, with 2,5-dihydroxybenzoic acid as the matrix.

**Synthesis.**

Conditions: **a**) Pd(PPh_3_)_4_,Na_2_CO_3_, dioxane, H_2_O, reflux; **b**) NaH, DMF, 70 - 150 °C; **c**) bis(pinacolato)diboron, CH_3_COOK, PdCl_2_(dppf), dioxane, reflux.

**Scheme S1.** Synthesis of **DMAC-diF-TRZ** and **DMAC-diCl-TRZ**

*General procedure for the Suzuki-Miyaura cross-coupling* (step a, Scheme S1)*.* The mixture of 2-chloro-4,6-diphenyl-*s*-triazine (1 g, 3.7 mmol), Pd(PPh_3_)_4_ (0.1 g, 0.09 mmol), 2 M aqueous K_2_CO_3_ (4 mL) in dioxane (20 mL), and 3,4,5-trifluorophenylboronic acid (0.66 g, 3.7 mmol) for **triF-TRZ** or **DMAC-diClPhBpin** (1.8 g, 3.7 mmol) for **DMAC-diCl-TRZ** was refluxed under Ar atmosphere for 12h. The reaction progress was monitored by TLC. After the completion, the solvent was evaporated under reduced pressure and the residue was treated with 0.1 M aqueous HCl, extracted with dichloromethane and purified by column chromatography.

*2-(3,4,5-Trifluorophenyl)-4,6-diphenyl-s-triazine* (**triF-TRZ**): white crystalline powder, yield 99%. ^1^H NMR (500 MHz, CDCl_3_, *δ*): 7.58–7.71 (m, 6H), 8.45 (m, 2H), 8.76 (d, 4H, *J* = 7.2 Hz). ^13^C NMR (250 MHz, CDCl_3_, *δ*): 172.0, 135.6, 133.0, 129.0, 128.8, 113.1. MALDI-TOF MS *m/z*: calcd for C_21_H_12_N_3_F_3_, 363.10 [*M*]^+^; found, 364.17 [*M +* H]^+^.

*10-(3,5-Dichloro-4-(4,6-Diphenyl-s-triazin-2-yl)phenyl)-9,9-dimethyl-9,10-dihydroacridine* (**DMAC-diCl-TRZ**): yellow crystalline powder, yield 67%. ^1^H NMR (500 MHz, CDCl_3_, *δ*): 1.80 (s, 6H), 6.21 (dd, 2H, *J* = 8.0 Hz, *J =* 1.4 Hz), 7.04 (td, 2H, *J* = 7.5 Hz, *J =* 1.8 Hz), 7.07 (td, 2H, *J* = 7.7 Hz, *J =* 1.8 Hz), 7.57 (dd, 2H, *J* = 7.6 Hz, *J =* 1.7 Hz), 7.62–7.71 (m, 6H), 8.82 (d, 4H, *J* = 7.9 Hz), 8.98 (s, 2H). ^13^C NMR (250 MHz, CDCl_3_, *δ*): 172.2, 169.2, 138.9, 138.7, 135.6, 133.1, 130.1, 130.0, 126.9, 126.2, 125.3, 121.5, 112.5, 36.0, 32.7. MALDI-TOF MS *m/z*: calcd for C_36_H_26_N_4_Cl_2_, 584.15 [*M*]^+^; found, 569.15 [*M –* CH_3_]^+^. Elem. anal. calcd, %: C 73.85; H 4.48; N 9.57. Found, %: C 73.90; H 4.52; N 9.50.

*General procedure for a nucleophilic aromatic substitution* (S_N_Ar, step b, Scheme S1). A solution of DMAC (1 g, 4.8 mmol) and NaH, 60% in mineral oil (0.19 g, 4.7 mmol) in dry dimethylformamide (DMF) was stirred under an inert atmosphere for 30 min. Then, **triF-TRZ** (1.65 g, 4.55 mmol) for **DMAC-diF-TRZ** or 5-bromo-1,3-dichloro-2-fluorobenzene (1.33 g, 5.5 mmol) for **DMAC-diClPhBr** was added, and the mixture was heated at 70 – 150 °C with continuous stirring. The reaction progress was monitored by thin-layer chromatography (TLC). Upon completion, the reaction mixture was cooled and the solvent evaporated under reduced pressure. The residue was treated with 0.1 M aqueous HCl, followed by extraction with dichloromethane and purification by silica gel column chromatography.

*10-(3,5-Difluoro-4-(4,6-diphenyl-s-triazin-2-yl)phenyl)-9,9-dimethyl-9,10-dihydroacridine* (**DMAC-diF-TRZ**): Yellow crystalline powder, yield 87%. ^1^H NMR (500 MHz, CDCl_3_, *δ*): 1.74 (s, 6H), 6.44 (d, 2H, *J* = 7.9 Hz), 7.03 (t, 2H, *J* = 7.5 Hz), 7.08 (t, 2H, *J* = 7.9 Hz), 7.53 (dd, 2H, *J* = 7.9 Hz; *J* = 1.4 Hz), 7.59–7.69 (m, 6H), 8.59 (d, 2H, *J* = 8.1 Hz), 8.80 (d, 4H, *J* = 7.9 Hz). ^13^C NMR (250 MHz, CDCl_3_, *δ*): 172.2, 138.9, 135.6, 133.1, 131.0, 129.1, 128.8, 126.8, 125.4, 121.8, 113.4, 113.2, 113.0, 36.2, 30.7. MALDI-TOF MS *m/z*: calcd for C_36_H_26_N_4_F_2_, 552.21 [*M*]^+^; found, 537.22 [*M –* CH_3_]^+^. Elem. Anal. Calcd, %: C 78.24; H 4.74; N 10.14. Found, %: C 78.19; H 4.78; N 10.08.

*10-(4-Bromo-2,6-dichlorophenyl)-9,9-dimethyl-9,10-dihydroacridine* (**DMAC-diClPhBr**): white crystals, yield 53%. ^1^H NMR (500 MHz, CDCl_3_, *δ*): 1.75 (s, 6H), 6.10 (d, 2H, *J* = 8.0 Hz), 6.99–7.08 (m, 4H), 7.53 (d, 2H, *J* = 7.5 Hz), 7.79 (s, 2H). ^13^C NMR (250 MHz, CDCl_3_, *δ*): 139.1, 137.2, 135.0, 132.7, 130.1, 126.9, 126.2, 122.5, 121.5, 112.3, 35.9, 32.6. MALDI-TOF MS *m/z*: calcd for C_21_H_16_BrCl_2_N, 432.98 [*M*]^+^; found, 417.99 [*M –* CH_3_]^+^.

*10-(4-(4,4,5,5-tetramethyl-1,3,2-dioxaborolan-2-yl)-2,6-dichlorophenyl)-9,9-dimethyl-9,10-dihydroacridine* (step c, Scheme S1*,* **DMAC-diClPhBpin**). A mixture of **DMAC-diClPhBr** (2.2 g, 5.1 mmol), bis(pinacolato)diboron (1.5 g, 5.9 mmol), dry potassium acetate (1.7 g, 17.8 mmol) and (1,1'-bis(diphenylphosphino)ferrocene)palladium(II) dichloride (0.11 g, 0.15 mmol), in dry dioxane (22 mL), was refluxed under stirring in Ar atmosphere for 10h. The reaction progress was monitored by TLC. After the completion, the solvent was evaporated under reduced pressure. The residue was then treated with water, extracted with dichloromethane and purified by column chromatography to give **DMAC-diClPhBpin** in 87% yield. Pale-yellow solid, ^1^H NMR (500 MHz, CDCl_3_, *δ*): 1.42 (s, 12H), 1.76 (s, 6H), 6.09 (d, 2H, *J* = 7.8 Hz), 6.96–7.05 (m, 4H), 7.52 (d, 2H, *J* = 7.4 Hz), 8.02 (s, 2H). ^13^C NMR (250 MHz, CDCl_3_, *δ*): 137.7, 137.4, 135.6, 130.0, 126.8, 126.0, 121.2, 112.5, 84.8, 35.9, 32.6, 24.9. MALDI-TOF MS *m/z*: calcd for C_27_H_28_BCl_2_NO_2_, 479.16 [*M*]^+^; found, 464.20 [*M –* CH_3_]^+^.

*Sample Preparation.* During the crystal grown by slow solvent evaporation from various solvents (toluene, ethyl acetate, dioxane, chloroform) **DMAC-diF-TRZ** and especially **DMAC-TRZ** exhibited rather complex solidification behavior. Both compounds formed multiple mostly small and needle-shaped crystal morphologies, with distinct emission colors ranging from blue to yellow or green, respectively, most likely reflecting differences in DMAC planarity and/or *θ*_DA_ in polymorph systems. In contrast, **DMAC-diCl-TRZ** showed highly reproducible crystallization behavior, yielding large, well-faceted, cuboid-like single crystals up to 5 × 5 × 3 mm^3^ in size (Figure 2, main text). This is an essential feature for reliable radioluminescence measurements (particularly for gamma spectroscopy) and applications, indicating that chlorine substitution promotes the formation of macroscopic, optically homogeneous crystals.

*Single-Crystal X-Ray Diffraction (SCXRD) measurements and structure refinement***.** SCXRD data were collected using a Rigaku SuperNova diffractometer equipped with a single Mo *K*_α_ microfocus X-ray source (λ = 0.71073 Å) and an Eos detector at T = 100(2) K (Table S1). Single crystals suitable for X-ray diffraction were obtained by slow evaporation from toluene (**DMAC-diF-TRZ)** or toluene-ethyl acetate mixture (**DMAC-diCl-TRZ).** The lattice parameters were obtained by least-squares fit to the optimised setting angles of the reflections collected by means of CrysAlis CCD. Data were reduced using CrysAlis RED software and applying multi-scan absorption corrections.^S[1]^ The structures were solved with direct methods that carried out refinements by full-matrix least-squares on *F*^2^ using the SHELXL-2017/1 program.^S[2]^ Structural analysis revealed the presence of solvent-accessible voids in the crystals of **DMAC-diCl-TRZ**. The solvent molecules occupying these voids exhibited severe static rotational disorder around crystallographic inversion centres, preventing reliable modelling with discrete atomic positions. The disordered solvent was treated using the Solvent Mask routine implemented in Olex2. The solvent mask identified two symmetry-equivalent solvent-accessible voids centred at (0.5, 0, 0.5) and (0.5, 0.5, 0). All H-atoms bound to C-atoms were placed geometrically and refined using a riding model with d_(C–H)_ = 0.93–0.97 Å and U_iso_(H) = 1.2U_eq_(C) (d_(C–H)_ = 0.96 Å and U_iso(_H) = 1.5U_eq_(C) for the -CH_3_ groups). The following programs were used to prepare the molecular graphics: ORTEPII, and Mercury.^S[3],S[4]^

*Photoluminescence (PL) measurements.* Steady-state PL spectra and absolute PL quantum were recorded using FS5 spectrofluorometer (Edinburgh Instruments) using front-face excitation geometry. Time-resolved PL measurements were performed at different temperatures using a customized system consisting of a pulsed YAG:Nd laser (PL2251A, EKSPLA) coupled with an optical parametric generator (PG 401/SH) as an excitation light source and 2501S grating spectrometer (Bruker Optics) combined with the streak camera system (C4334-01 Hamamatsu) as a detection unit.^S^^[5]^ The system was equipped with a double-stage high vacuum pump (T-Station 85 Edwards) coupled with a closed-cycle helium cryostat (APD DE-202) and the temperature controller (LakeShore 336). PL intensity decay profiles were obtained by integration of streak camera images over constant wavelength interval using several time windows from 20 ns to 40 ms.

*Quantum chemical calculations.* Density functional theory (DFT) calculations were performed without geometry optimization, using crystal geometries obtained from X-ray diffraction analysis. All calculations were carried out at the TDDFT level of theory within the Tamm-Dancoff approximation (TDA) using ORCA program package, ver. 6.1.^S^^[6]^ The CAM-B3LYP^S^^[7]^ and M06-2X^S^^[8]^ functionals were used with the def2-SVP basis set for all atoms, except for chlorine, for which the def2-TZVP basis set was applied, including the relativistic zero-order regular approximation (ZORA).

*Radioluminescence (RL) measurements.* A typical set-up consisting of an Inel XRG3500 X-ray generator (Cu-anode tube, 45 kV / 10 mA), an Acton Research Corporation SpectraPro-500i monochromator (500 nm blazed grating), a Hamamatsu R928 photomultiplier, and an APD Cryogenics Inc. closed-cycle helium cooler with a Lake Shore 330 programmable temperature controller, was used to record radioluminescence spectra at various temperatures between 10 and 350 K. We note that the measurements were carried out starting at 350 K and terminating at 10 K to avoid a possible contribution from thermal release of charge carriers to the emission yield. The same setup was employed to measure a low temperature thermoluminescence (ltTL) glow curve. Prior to the ltTL run, the sample was exposed for 10 min to X-rays at 10 K. The glow curve was recorded between 10 and 350 K at a heating rate of about 0.08 K/s.

For X-Ray imaging the scintillation screen (dimensions approx.1.5 cm x 1 cm) was prepared by uniformly mechanical distribution of **DMAC-diCl-TRZ** powder onto the surface of a transparent substrate. The resulting image of the object (small chip, 3 mm wide) was captured using Basler acA1300-200um camera.

*Gamma spectroscopy measurements.* Pulse height spectra (PHS) were measured at room temperature under 59.5 keV and 662 keV gamma excitation from an ^241^Am and a ^137^Cs source, respectively. The pulsed output signal from a Hamamatsu R878 photomultiplier (PMT) was processed by a Canberra 2005 integrating preamplifier, a Canberra 2022 spectroscopy amplifier (shaping time of 8 μs), and a Tukan 8k USB multichannel analyzer. To improve the light collection efficiency, the sample was coupled to the PMT window with Viscasil grease and covered with several layers of Teflon tape.

Scintillation time profiles (STP) were taken by the delayed coincidence single photon counting method.^S^^[9]^ The sample was excited by gamma rays from a ^137^Cs source and time profiles of subsequent emission pulses were registered by collecting a large number of scintillation photons and recording their time distribution. Two Hamamatsu PMTs (R2059 for “starts” / R928 for “stops”; HV of 1790 and 1190 V, respectively), a Canberra TAC/SCA 2145 time-to-amplitude converter, and a Tukan 8k USB multichannel analyzer were utilized.

*Details of determination of the scintillation light yield.* Based on the full-energy peak positions, the numbers of photoelectrons released from the photocathode per 1 MeV of energy deposited in the crystal were calculated. Subsequently, the values of scintillation yield were estimated considering the spectral matching of the investigated sample to the PMT characteristics. The so-called photoelectron yield is calculated using the following equation:

$$Y=\frac{PP\cdot G_{SPP}}{SPP\cdot G_{PP}\cdot E_{exc}}$$

where PP denotes the position of the full-energy peak, SPP - the position of the single-photoelectron peak, G_PP_ - the amplifier gain used during the measurement with the sample, G_SPP_ - the amplifier gain used during the single-photoelectron measurement, and E_exc_ - the excitation energy of the radiation source (59.5 keV or 662 keV in the present study). The result, however, is expressed in units of photoelectrons per 1 MeV. To convert it into the units of photons per 1 MeV, we assume a very high reflectivity of the PTFE reflector and just divide the photoelectron yield by the effective quantum efficiency of the photomultiplier tube, which accounts for the spectral matching between the photomultiplier characteristics and the radioluminescence emission spectrum of the investigated sample.

Spectral characteristics of the R878 PMT are given below:

*Statistical Analysis.* SCXRD data were processed and refined using standard crystallographic procedures. Lattice parameters were obtained by least-squares fitting, and the structures were refined by full-matrix least-squares on F^2^. Complete data-collection and refinement statistics are provided in Table S1. Arrhenius analysis was performed by linear regression of ln(*k*_rISC_) versus 1/T within the temperature range showing linear behavior. The uncertainties of *E*_a_ were calculated by propagation of the standard error of the fitted slope; calculations were carried out using Origin Pro software.

**Single crystal XRD analyses**

**Table S1.** Crystal data and structure refinement for **DMAC-diF-TRZ** and **DMAC-diCl-TRZ**

| **Compound** | | **DMAC-diF-TRZ** | **DMAC-diCl-TRZ** |
| --- | --- | --- | --- |
| Chemical formula | | C_36_H_26_N_4_F_2_ | C_36_H_26_N_4_Cl_2_ [+ solvent] |
| Formula weight/g·mol^-1^ | | 552.61 | 585.51 |
| Crystal system | | triclinic | monoclinic |
| Space group | | *P*$\overline{1}$ | *P*2_1_/c |
| a/Å | | 9.4325(6) | 9.2473(3) |
| b/Å | | 10.8771(8) | 27.7918(7) |
| c/Å | | 14.1226(9) | 12.4171(4) |
| α/° | | 92.181(6) | 90 |
| β/° | | 97.367(5) | 101.501(3) |
| γ/° | | 109.067(6) | 90 |
| V/Å^3^ | | 1353.20(16) | 3127.11(17) |
| Z | | 2 | 4 |
| T/K | | 100 | 100 |
| Λ_Cu_/Å | | 0.71073 | 0.71073 |
| ρ_calc_/g·cm^–3^ | | 1.356 | 1.244 |
| F(000) | | 576 | 1216 |
| µ/mm^-1^ | | 0.090 | 0.239 |
| θ range/° | | 3.39 - 26.37 | 3.35 - 26.37 |
| Completeness θ/% | | 99.8 | 99.8 |
| Reflections collected | | 17425 | 19571 |
| Reflections unique | | 5525 [R_int_=0.0983] | 6386 [R_int_=0.0599] |
| Data/restraints/parameters | | 5525 / 0 / 381 | 6386 / 0 / 381 |
| Goodness of fit on F^2^ | | 1.176 | 1.066 |
| Final R_1_ value (I>2σ(I)) | | 0.0623 | 0.0469 |
| Final wR_2_ value (I>2σ(I)) | 0.1820 | | 0.1195 |
| Final R_1_ value (all data) | 0.0743 | | 0.0625 |
| Final wR_2_ value (all data) | 0.2118 | | 0.1295 |
| Largest diff. peak/hole/e Å^−3^ | 0.461/-0.354 | | 0.284/-0.426 |
| CCDC number | 2543421 | | 2543422 |
| Molecular structure with the atom-label-ling (displacement ellipsoids are drawn at the 25% probability level; hydrogen atoms are drawn as spheres of arbitrary radii) |  | |  |

(a)

(b)

**Figure S1.** Molecular structures of **DMAC-TRZ**, **DMAC-diCl-TRZ**, and **DMAC-diF-TRZ** viewed (a) perpendicular to and (b) along the N1–C16 bond.

**Figure S2.** Molecular structures of acceptor–acceptor (A–A, left) and donor–donor (D–D, right) dimers.

*Additional discussion on structure-property relationships.* Single-crystal X-ray diffraction analysis revealed that **DMAC-diF-TRZ** crystallizes in the triclinic *P*–1 space group, whereas **DMAC-diCl-TRZ** crystallizes as a solvate in the monoclinic P2₁/c space group and is isostructural with DMAC-TRZ solvate^S^^[10]^ (Table S1). All compounds exhibit different donor geometries and packing motifs. **DMAC-diF-TRZ** contains the most strongly bent and asymmetric DMAC fragment, with two distinct D–A dihedral angles of 81.6° and 75.8°, and uniquely forms both A–A and D–D stacking motifs. In contrast, **DMAC-diCl-TRZ** exhibits a substantially more planar donor and an almost perfectly orthogonal D–A geometry (θ_DA_= 87.0°) together with A–A stacking and characteristic Cl···Cl, Cl···H, and Cl···C contacts. The parent compound occupies an intermediate structural regime.

Structural changes in the linker region are less pronounced. The angles between the phenylene linker and the triazine unit are comparable for the three compounds, with values of 10.7°, 5.8°, and 7.7°, respectively. However, a view along the N1–C16 bond reveals that the triazine fragment in **DMAC-diF-TRZ** is rotated by approximately 180° relative to the difluorophenyl ring (Figure S1b), further emphasizing that fluorination and chlorination do not produce simple incremental structural changes.

As specifically discussed through the main text, from the point of view of structure-property relationships, these structural differences correlate with the observed photo-physical behavior. In particular, the D–D interactions present only in the fluorinated cry-stal stabilize the lowest singlet state and contribute to the red-shifted emission, whereas the slipped A–A motifs of the parent and chlorinated crystals produce different energetic effects. In **DMAC-diCl-TRZ**, the rigid donor geometry and Cl-specific intermolecular contacts support efficient triplet harvesting by combining a small effective rISC barrier with dynamically accessible SOC channels. From the perspective of engineering crystal-phase TADF scintillators, the structural consequences of F and Cl substitution are funda-mentally different. **DMAC-diF-TRZ** contains the most strongly bent donor and uniquely forms D–D aggregates, which stabilize the S_1_ state and cause the red-shifted emission, but simultaneously weaken LE–CT spin–orbit coupling, resulting in only moderately high scintillation LY. In contrast, **DMAC-diCl-TRZ** owes its remarkable triplet-harvesting efficiency and scintillation performance to the combination of a rigid, planar donor and asymmetric intermolecular contacts that modulate chlorine motion within the crystal.

**Additional photoluminescence and radioluminescence data**

**Figure** **S3**. Time-resolved emission spectra (TRES) at various temperatures for **DMAC-diF-TRZ**

**Determination of activation energy *E*_a_**

Arrhenius analysis was performed by linear regression of ln(*k*_rISC_) versus 1/T within the temperature range showing linear behavior. For **DMAC-TRZ**, the fit was restricted to 150–300 K, whereas for **DMAC-diF-TRZ** and **DMAC-diCl-TRZ**, the range of 30–300 K was used. Data outside these ranges were excluded from the linear fit because of pronounced deviations from Arrhenius behavior, attributed to additional low-temperature emission processes, probably phosphorescence, competing with TADF. Activation energies were obtained from the fitted slope, and their uncertainties were calculated by propagation of the standard error of the slope.

Activation energies (*E*_a_) of studied compounds were determined by Arrhenius formula:

$$k_{rISC}=Ae^{\left( - \frac{E_{a}}{k_{B}T} \right)}$$

The linearization of this equation allows for the application of linear regression analysis:

$${ln(k}_{rISC})=\ln\left( A \right)- \frac{E_{a}}{k_{B}} \left( \frac{1}{T} \right),$$

where:

$$a=- \frac{E_{a}}{k_{B}}$$

$$E_{a}=- k_{B} a$$

Given that E_a_ is calculated as a derived function, its uncertainty ∆*E*_a_ was estimated using the law of error propagation via the total differential method:

$${\Delta E}_{a}=\left| \frac{\partial E_{a}}{\partial a} \right|\cdot\Delta a= k_{B}\cdot\Delta a$$

Summary of Arrhenius fitting parameters and derived activation energies are given below:

| **Crystal** | **Number of points** | **R^2^** | **Slope** | **Std. Error** | ***E*_a_ [meV]** | **∆*E*_a_ [meV]** |
| --- | --- | --- | --- | --- | --- | --- |
|  | **n** |  | **a** | **∆a** |  |  |
| **DMAC-TRZ** | 16 | 0.98 | -1773.1 | 15.8 | 153 | 2 |
| **DMAC-diF-TRZ** | 14 | 0.95 | -85.9 | 4.1 | 7.1 | 0.4 |
| **DMAC-diCl-TRZ** | 11 | 0.93 | -45.6 | 5.5 | 3.9 | 0.5 |

**Figure S4**. Temperature-dependent PL emission decay curves of the studied crystals (top) and full-range dependence of ln(k_rISC_) on 1/*T* (bottom). The linear regions used for the Arrhenius fits are indicated.

**Table S2.** Experimental data used for Arrhenius analysis for **DMAC-TRZ**

| T [K] | 1/T [K^-1^] | *k*_rISC_ [10^4^ s^-1^] | ln(*k*_rISC_) |
| --- | --- | --- | --- |
| 300 | 0.0033 | 5.427 | 10.90 |
| 290 | 0.0034 | 4.253 | 10.66 |
| 280 | 0.0036 | 3.234 | 10.38 |
| 270 | 0.0037 | 2.554 | 10.15 |
| 260 | 0.0038 | 1.965 | 9.89 |
| 250 | 0.0040 | 1.573 | 9.66 |
| 240 | 0.0042 | 1.116 | 9.32 |
| 230 | 0.0043 | 0.745 | 8.92 |
| 220 | 0.0045 | 0.566 | 8.64 |
| 210 | 0.0048 | 0.383 | 8.25 |
| 200 | 0.0050 | 0.269 | 7.90 |
| 190 | 0.0053 | 0.157 | 7.36 |
| 180 | 0.0056 | 0.085 | 6.74 |
| 170 | 0.0059 | 0.054 | 6.29 |
| 160 | 0.0063 | 0.031 | 5.75 |
| 150 | 0.0067 | 0.014 | 4.94 |

**Table S3.** Experimental data used for Arrhenius analysis for **DMAC-diF-TRZ**

| T [K] | 1/T [K^-1^] | *k*_rISC_ [10^4^ s^-1^] | ln(*k*_rISC_) |
| --- | --- | --- | --- |
| 300 | 0.0033 | 244.2 | 14.71 |
| 260 | 0.0038 | 225.5 | 14.63 |
| 240 | 0.0042 | 204.0 | 14.53 |
| 220 | 0.0045 | 200.3 | 14.51 |
| 200 | 0.0050 | 198.6 | 14.50 |
| 180 | 0.0056 | 189.3 | 14.45 |
| 160 | 0.0063 | 188.1 | 14.45 |
| 120 | 0.0083 | 161.3 | 14.29 |
| 100 | 0.0100 | 114.0 | 13.95 |
| 80 | 0.0125 | 88.1 | 13.69 |
| 60 | 0.0167 | 67.1 | 13.42 |
| 50 | 0.0200 | 43.9 | 12.99 |
| 40 | 0.0250 | 33.2 | 12.71 |
| 30 | 0.0333 | 20.9 | 12.25 |

**Table S4.** Experimental data used for Arrhenius analysis for **DMAC-diCl-TRZ**

| T [K] | 1/T [K^-1^] | *k*_rISC_ [10^4^ s^-1^] | ln(*k*_rISC_) |
| --- | --- | --- | --- |
| 300 | 0.0033 | 457.64 | 15.34 |
| 160 | 0.0063 | 438.18 | 15.29 |
| 140 | 0.0071 | 412.26 | 15.23 |
| 120 | 0.0083 | 395.58 | 15.19 |
| 100 | 0.0100 | 330.35 | 15.01 |
| 80 | 0.0125 | 256.07 | 14.76 |
| 60 | 0.0167 | 225.20 | 14.63 |
| 50 | 0.0200 | 190.83 | 14.46 |
| 40 | 0.0250 | 170.91 | 14.35 |
| 30 | 0.0333 | 130.39 | 14.08 |

**Table S5.** Radioluminescence and scintillation properties of **DMAC-diCl-TRZ** crystal*

| **Compound** | **LY_59.5 keV_ [ph/MeV]** | **LY_662 keV_ [ph/MeV]** | ***τ*_PF_^sc^ [ns]** | ***f*_PF_^sc^ [%]** | ***τ*_DF_^sc^ [μs]** | ***f*_DF_^sc^ [%]** |
| --- | --- | --- | --- | --- | --- | --- |
| **DMAC-diCl-TRZ** | 22 900 | 26 000 | 10.8 | 2 | 1.8 | 98 |

*LY at 59.5 keV was determined from the full-energy peak under ^241^Am excitation. LY at 662 keV and decay parameters were estimated under ^137^Cs excitation.

**A	B**

|  |  |
| --- | --- |
| **C** | |
|  | |

**Figure S5**. **DMAC-diCl-TRZ** : (A) ^137^Cs pulse-height spectra of **DMAC-diCl-TRZ** crystals . (B) Two-dimensional map of X-ray excited RL spectra of recorded over the temperature range 10-350 K. (C) Low temperature thermoluminescence.

**Operational reproducibility test**

**Figure S6.** Operational reproducibility of **DMAC-diCl-TRZ** under repeated X-ray excitation. Time-dependent RL intensity recorded during stepwise variation of X-ray tube voltage (15-45 kV) and current (6-10 mA).

**X-ray attenuation coefficients**

**Figure S7.** Mass attenuation coefficients of **DMAC-TRZ**, **DMAC-diF-TRZ**, **DMAC-diCl-TRZ**, and anthracene as a function of photon energy. Element-specific absorption edges are indicated. The inset enlarges the low-energy region containing the N and F K-edges.

Figure S7 shows the theoretical mass attenuation coefficients (µ_m_) of **DMAC-TRZ, DMAC-diF-TRZ, DMAC-diCl-TRZ**, and anthracene obtained using the NIST XCOM database.^S^^[11]^ All samples exhibit the expected strong energy dependence, with a gradual decrease as photon energy increases due to the reduced probability of photoelectric absorption. Distinct discontinuities observed correspond to elemental absorption edges including the K-edge of carbon at 0.28 keV, which is common to all compounds and K-edge of nitrogen at 0.40 keV. Additional absorption edges at 0.69 keV and 2.82 keV are attributed to fluorine and chlorine K-shell ionization in **DMAC-diF-TRZ** and **DMAC-diCl-TRZ**, respectively, while the chlorine L-edge appears near 0.20 keV; all edge energy values were matched with literature values reported in [^S^^[12]^]. Among all samples, **DMAC-diCl-TRZ** exhibits the highest attenuation over a wider energy range, whereas anthracene shows the lowest attenuation because of its lower effective atomic number and absence of heteroatoms. At higher energies, all curves gradually converge as Compton scattering becomes the dominant interaction mechanism.

**Calculations**

**Figure S8**. Energy alignment of excited states with significant SOC shown for the monomer species, geometries extracted from XRD analysis (M06-2X/def2-SVP).

**General comment.** Computational analysis was performed using geometries from the XRD structures, without geometry optimization to make sure the key structural features remain close to the experimental conditions. For this reason, direct comparison between the calculated vertical excitation energies and the experimental emission energies should be made with caution. This limitation is particularly important for D-A TADF systems, where even small changes in donor planarity, *θ*_DA_, local polarity, or intermolecular interactions can cause large changes in CT-state energies. All three compounds also differ in donor geometry and crystal packing, so a direct numerical comparison of very small energy differences between them is especially demanding for the applied DFT methods. The fluorinated and chlorinated derivatives are also not identical systems, specifically regarding structural parameters. The substitution changes donor geometry, molecular symmetry, intermolecular contacts, CT character, and the distribution of excited states. In addition, the XRD geometries are experimental ground-state structures and are not optimized for a given functional.

**Table S6**. Summary of the calculated parameters of key excited states for various species (M06-2X/def2-SVP)

| **Cmpd** | **Species** | ***E*_S1_ [eV]** | **number of triplet** | ***E*_Tn_**  **[eV]** | **Δ*E*_S1-Tn_ [eV]** | **S_1_-T_n_ SOC [cm^-1^]** |
| --- | --- | --- | --- | --- | --- | --- |
| **DMAC-TRZ** | **monomer** | **3.465** | 1 | 3.455 | 0.010 | 0.08 |
|  |  |  | 2 | 3.713 | -0.25 | 0.68 |
|  |  |  | 3 | 3.83 | -0.37 | 0.87 |
|  |  |  | 4 | 3.941 | -0.48 | 0.94 |
|  | **dimer A-A** | **3.56** | 1 | 3.549 | 0.011 | 0.11 |
|  |  |  | 3 | 3.689 | -0.13 | 0.52 |
|  |  |  | 6 | 3.811 | -0.25 | 0.85 |
|  |  |  | 9 | 3.903 | -0.34 | 0.68 |
|  |  |  |  |  |  |  |
| **DMAC-diF-TRZ** | **monomer** | **3.485** | 1 | 3.466 | 0.019 | 0.13 |
|  |  |  | 2 | 3.67 | -0.19 | 0.24 |
|  |  |  | 4 | 3.984 | -0.50 | 0.77 |
|  | **dimer A-A** | **3.554** | 1 | 3.532 | 0.022 | 0.15 |
|  |  |  | 4 | 3.643 | -0.09 | 0.20 |
|  |  |  | 7 | 3.948 | -0.39 | 0.35 |
|  |  |  | 9 | 4.032 | -0.48 | 0.54 |
|  | **dimer D-D** | **3.409** | 1 | 3.393 | 0.016 | 0.10 |
|  |  |  | 4 | 3.673 | -0.26 | 0.27 |
|  |  |  | 7 | 3.968 | -0.56 | 0.73 |
|  |  |  | 9 | 3.98 | -0.57 | 0.62 |
|  | **trimer** | **3.402** | 2 | 3.386 | 0.016 | 0.10 |
|  |  | **S2** | 5 | 3.64 | -0.24 | 0.20 |
|  |  |  | 11 | 3.943 | -0.54 | 0.68 |
|  |  |  | 14 | 3.983 | -0.58 | 0.56 |
|  |  |  |  |  |  |  |
| **DMAC-diCl-TRZ** | **monomer** | **3.169** | 1 | 3.160 | 0.009 | 0.04 |
|  |  |  | 2 | 3.689 | -0.52 | 0.48 |
|  |  |  | 3 | 3.897 | -0.73 | 0.46 |
|  |  |  | 4 | 3.906 | -0.74 | 0.99 |
|  | **dimer A-A** | **3.262** | 1 | 3.253 | 0.009 | 0.02 |
|  |  |  | 4 | 3.658 | -0.40 | 0.37 |
|  |  |  | 5 | 3.838 | -0.58 | 0.50 |
|  |  |  | 8 | 3.881 | -0.62 | 1.08 |
|  | **dimer Cl-Cl** | **3.206** | 1 | 3.196 | 0.010 | 0.02 |
|  |  |  | 4 | 3.691 | -0.49 | 0.45 |
|  |  |  | 6 | 3.899 | -0.69 | 0.48 |
|  |  |  | 8 | 3.905 | -0.70 | 0.93 |
|  | **tetramer** | **3.341** | 4 | 3.331 | 0.01 | 0.02 |
|  | **Cl-H** | **S4** | 12 | 3.689 | -0.35 | 0.94 |
|  |  |  | 26 | 3.905 | -0.56 | 3.88 |

**Complementary CAM-B3LYP analysis.** To check how strongly the calculated excited-state properties depend on the applied functional, all monomers and selected aggregate species were additionally calculated using CAM-B3LYP (Table S7).

Regarding the prediction energies of the S_1_ states and the S_1_-T_1_ energy gaps for **DMAC-TRZ, DMAC-diF-TRZ,** and **DMAC-diCl-TRZ,** the CAM-B3LYP predicted values give worse correlation with the experimentally observed trends. However, the comparison of relative changes caused by aggregation lead to generally similar conclusions as made based on the M062X calculations. Formation of the **DMAC-TRZ** A-A dimer increases the S_1_ energy by 95 meV. In **DMAC-diF-TRZ**, such a dimer raises the S_1_ energy by 71 meV, whereas the D···D–A···A trimer lowers it by 122 meV relative to the monomer. This result supports the conclusion that donor-donor interactions are responsible for stabilization of the lowest singlet state in the fluorinated crystal. For **DMAC-diCl-TRZ**, formation of the Cl···Cl dimer changes the S_1_ energy by only 37 meV, whereas the singlet state localized on the molecule involved in the Cl···H environment of the tetramer is shifted to higher energy by about 168 meV. Thus, despite differences in absolute energies, both M06-2X and CAM-B3LYP predict similar effects of the characteristic intermolecular contacts on the singlet states.

Larger differences between the two functionals are found for Δ*E*_S1T1_. According to CAM-B3LYP, theses values are quite large for the **DMAC-TRZ** monomer and dimer reaching 133 and 235 meV, respectively. For **DMAC-diF-TRZ**, the corresponding values are even larger: 177 meV for the monomer and 282 meV for the A-A dimer, but the it decreases to 91 meV in the D···D–A···A trimer. Thus, CAM-B3LYP does not reproduce the very small experimental activation energy of **DMAC-diF-TRZ**, but it does support the conclusion that D-D interactions reduce the singlet-triplet separation. For **DMAC-diCl-TRZ**, the monomer and dimer give relatively small Δ*E*_S1T1_ of about 20 meV, whereas the corresponding separation for the locally relevant state in the tetramer is larger. The exact value therefore depends strongly on the molecular environment, confirming that a simple comparison of one calculated energy gaps with the experimental activation energy is not sufficient for these systems.

The key advantage of CAM-B3LYP calculations is apparently more correct predition of the alignment of the higher triplet states of LE nature. Where M06-2X calculations predict relatively large SOC with the triplet states separated from the relevant singlet by more than 0.3 eV, CAM-B3LYP places several triplet states much closer in energy. For example, the **DMAC-TRZ** dimer contains several triplets within 5-74 meV of S_1_, with SOC values reaching 0.79 cm^-1^. In **DMAC-diF-TRZ**, relevant triplet states occur 35-71 meV from the singlet states, with SOC values up to 0.48 cm^-1^, while the trimer contains a channel separated by 66 meV with SOC = 0.34 cm^-1^. In the **DMAC-diCl-TRZ** tetramer, a triplet state is found 57 meV from the relevant singlet state with SOC = 0.59 cm^-1^, together with additional close-lying states.

**Table S7.** Summary of calculated parameters for energetically proximate singlet-triplet channels in the studied species (CAM-B3LYP/def2-SVP).

| **Compound** | **Species** | **S_n_** | **E_Sn_ [eV]** | **T_n_** | ***E*_Tn_**  **[eV]** | **Δ*E*_S1-Tn_**  **[eV]** | **S_1_-T_n_ SOC**  **[cm^-1^]** |
| --- | --- | --- | --- | --- | --- | --- | --- |
| **DMAC-TRZ** | **monomer** | S1 | 3.600 | T1 | 3.467 | 0.133 | 0.66 |
|  |  |  |  | T3 | 3.636 | -0.036 | 0.81 |
|  |  |  |  | T4 | 3.701 | -0.101 | 0.87 |
|  | **dimer A-A** | S1 | 3.695 | T1 | 3.460 | 0.235 | 0.50 |
|  |  |  |  | T4 | 3.621 | 0.074 | 0.79 |
|  |  |  |  | T5 | 3.658 | 0.037 | 0.45 |
|  |  |  |  | T6 | 3.661 | 0.034 | 0.35 |
|  |  |  |  | T7 | 3.700 | -0.005 | 0.45 |
|  |  |  |  | T8 | 3.704 | -0.009 | 0.39 |
| **DMAC-diF-TRZ** | **monomer** | S1 | 3.604 | T1 | 3.427 | 0.177 | 0.31 |
|  |  |  |  | T2 | 3.569 | 0.035 | 0.24 |
|  |  |  |  | T4 | 3.746 | -0.142 | 0.69 |
|  | **dimer A-A** | S1 | 3.675 | T1 | 3.393 | 0.282 | 0.11 |
|  |  |  |  | T4 | 3.620 | 0.055 | 0.30 |
|  |  |  |  | T7 | 3.744 | -0.069 | 0.28 |
|  |  |  |  | T8 | 3.746 | -0.071 | 0.48 |
|  |  |  |  | T9 | 3.797 | -0.122 | 0.20 |
|  |  |  |  | T10 | 3.797 | -0.122 | 0.65 |
|  | **trimer** | S1 | 3.482 | T1 | 3.391 | 0.091 | 0.00 |
|  |  |  |  | T3 | 3.416 | 0.066 | 0.34 |
| **DMAC-diCl-TRZ** | **monomer** | S1 | 3.292 | T1 | 3.272 | 0.020 | 0.14 |
|  |  |  |  | T2 | 3.444 | -0.152 | 0.71 |
|  | **dimer Cl-Cl** | S1 | 3.329 | T1 | 3.308 | 0.021 | 0.03 |
|  |  |  |  | T4 | 3.445 | -0.116 | 0.67 |
|  | **tetramer Cl-H** | S4 | 3.460 | T1 | 3.235 | 0.225 | 0.00 |
|  |  |  |  | T4 | 3.403 | 0.057 | 0.59 |
|  |  |  |  | T8 | 3.460 | 0.000 | 0.29 |
|  |  |  |  | T9 | 3.614 | -0.154 | 0.40 |
|  |  |  |  | T10 | 3.626 | -0.166 | 0.41 |

**Table S8.** Calculated parameters of several excited states for various species of **DMAC-diCl-TRZ** under deviation of valence angle ∠C-C-Cl by +-10° (M06-2X/def2-SVP)

| **∠C-C-Cl [**°**]** | **S_n_** | ***E*_S1_**  **[eV]** | **T_n_** | ***E*_Tn_**  **[eV]** | | | **Δ*E*_S1-Tn_ [eV]** | | **S_1_-T_n_ SOC [cm^-1^]** | |
| --- | --- | --- | --- | --- | --- | --- | --- | --- | --- | --- |
| **Cl-Cl dimer** | | | | | | | | | | |
| **optimal** | S1 | 3.206 | 1 | 3.196 | | | -0.01 | | 0.02 | |
|  | S1 | 3.206 | 2 | 3.196 | | | -0.01 | | 0.02 | |
|  | S1 | 3.206 | 3 | 3.691 | | | 0.485 | | 0.02 | |
|  | S1 | 3.206 | 4 | 3.691 | | | 0.485 | | 0.45 | |
|  | S1 | 3.206 | 5 | 3.899 | | | 0.693 | | 0.01 | |
|  | S1 | 3.206 | 6 | 3.899 | | | 0.693 | | 0.48 | |
|  | S1 | 3.206 | 7 | 3.905 | | | 0.699 | | 0.32 | |
|  | S1 | 3.206 | 8 | 3.905 | | | 0.699 | | 0.93 | |
|  | S1 | 3.206 | 9 | 3.94 | | | 0.734 | | 0.03 | |
|  | S1 | 3.206 | 10 | 4.00 | | | 0.794 | | 0.84 | |
| **1Cl down** | | | | | | | | |  | |
| +10 | S2 | 3.231 | T04 | 3.702 | | | 0.471 | | 0.94 | |
|  | S1 | 3.203 | T03 | 3.691 | | | 0.488 | | 0.46 | |
|  | S2 | 3.231 | T05 | 3.899 | | | 0.668 | | 0.99 | |
|  | S2 | 3.231 | T08 | 3.913 | | | 0.682 | | 0.81 | |
|  | S1 | 3.203 | T06 | 3.9 | | | 0.697 | | 0.46 | |
|  | S1 | 3.203 | T07 | 3.906 | | | 0.703 | | 0.99 | |
|  | S2 | 3.231 | T10 | 3.941 | | | 0.71 | | 0.97 | |
|  | S1 | 3.203 | T09 | 3.939 | | | 0.736 | | 0.82 | |
|  | S2 | 3.231 | T13 | 4.001 | | | 0.77 | | 0.51 | |
|  | S2 | 3.231 | T15 | 4.023 | | | 0.792 | | 2.44 | |
|  | S1 | 3.203 | T12 | 3.999 | | | 0.796 | | 0.29 | |
|  | S1 | 3.203 | T14 | 4.009 | | | 0.806 | | 0.29 | |
|  | S2 | 3.231 | T17 | 4.048 | | | 0.817 | | 1.22 | |
|  | S1 | 3.203 | T16 | 4.043 | | | 0.84 | | 3.12 | |
|  | S1 | 3.203 | T18 | 4.053 | | | 0.85 | | 3.35 | |
|  | S2 | 3.231 | T19 | 4.113 | | | 0.882 | | 0.66 | |
|  | S1 | 3.203 | T20 | 4.115 | | | 0.912 | | 1.11 | |
| **1Cl up** | | | | | | | | | | |
| -10 | S2 | 3.21 | T04 | 3.69 | | | 0.48 | | 0.44 | |
|  | S1 | 3.187 | T03 | 3.674 | | | 0.487 | | 2.34 | |
|  | S2 | 3.21 | T06 | 3.897 | | | 0.687 | | 0.54 | |
|  | S2 | 3.21 | T07 | 3.904 | | | 0.694 | | 0.98 | |
|  | S1 | 3.187 | T05 | 3.882 | | | 0.695 | | 3.10 | |
|  | S1 | 3.187 | T08 | 3.917 | | | 0.73 | | 0.99 | |
|  | S2 | 3.21 | T10 | 3.941 | | | 0.731 | | 0.88 | |
|  | S1 | 3.187 | T09 | 3.938 | | | 0.751 | | 0.84 | |
|  | S2 | 3.21 | T13 | 4.000 | | | 0.79 | | 0.41 | |
|  | S1 | 3.187 | T14 | 4.008 | | | 0.821 | | 0.25 | |
|  | S2 | 3.21 | T15 | 4.037 | | | 0.827 | | 4.13 | |
|  | S2 | 3.21 | T17 | 4.052 | | | 0.842 | | 2.01 | |
|  | S1 | 3.187 | T16 | 4.05 | | | 0.863 | | 5.70 | |
|  | S1 | 3.187 | T18 | 4.06 | | | 0.873 | | 5.06 | |
|  | S2 | 3.21 | T20 | 4.117 | | | 0.907 | | 0.97 | |
|  | S1 | 3.187 | T19 | 4.116 | | | 0.929 | | 1.95 | |
| **1Cl down, 1'Cl up** | | | | | | | | | | |
| +10, -10 | S1 | 3.186 | T03 | 3.675 | | | 0.489 | | 2.29 | |
|  | S1 | 3.186 | T05 | 3.883 | | | 0.697 | | 3.03 | |
|  | S1 | 3.186 | T08 | 3.918 | | | 0.732 | | 0.98 | |
|  | S1 | 3.186 | T09 | 3.938 | | | 0.752 | | 0.82 | |
|  | S1 | 3.186 | T13 | 4.005 | | | 0.819 | | 0.26 | |
|  | S1 | 3.186 | T17 | 4.051 | | | 0.865 | | 4.85 | |
|  | S1 | 3.186 | T18 | 4.062 | | | 0.876 | | 5.83 | |
|  | S1 | 3.186 | T20 | 4.116 | | | 0.93 | | 2.08 | |
|  | S2 | 3.236 | T04 | 3.702 | | | 0.466 | | 0.94 | |
|  | S2 | 3.236 | T06 | 3.898 | | | 0.662 | | 0.98 | |
|  | S2 | 3.236 | T07 | 3.912 | | | 0.676 | | 0.76 | |
|  | S2 | 3.236 | T10 | 3.942 | | | 0.706 | | 1.02 | |
|  | S2 | 3.236 | T12 | 3.999 | | | 0.763 | | 0.54 | |
|  | S2 | 3.236 | T14 | 4.016 | | | 0.78 | | 2.50 | |
|  | S2 | 3.236 | T16 | 4.049 | | | 0.813 | | 1.09 | |
|  | S2 | 3.236 | T19 | 4.115 | | | 0.879 | | 0.64 | |
|  | S4 | 4.022 | T07 | 3.912 | | | -0.11 | | 0.30 | |
|  | S4 | 4.022 | T14 | 4.016 | | | -0.006 | | 0.59 | |
| **Two Cl down** | | | | | | | | | | |
| 2x +10 | S1 | 3.228 | T04 | 3.702 | | | 0.474 | | 0.95 | |
|  | S1 | 3.228 | T05 | 3.899 | | | 0.671 | | 0.90 | |
|  | S1 | 3.228 | T06 | 3.899 | | | 0.671 | | 0.40 | |
|  | S1 | 3.228 | T08 | 3.914 | | | 0.686 | | 0.85 | |
|  | S1 | 3.228 | T10 | 3.94 | | | 0.712 | | 0.95 | |
|  | S1 | 3.228 | T11 | 3.998 | | | 0.77 | | 0.45 | |
|  | S1 | 3.228 | T14 | 4.009 | | | 0.781 | | 0.30 | |
|  | S1 | 3.228 | T16 | 4.026 | | | 0.798 | | 2.40 | |
|  | S1 | 3.228 | T17 | 4.047 | | | 0.819 | | 1.28 | |
|  | S1 | 3.228 | T20 | 4.113 | | | 0.885 | | 0.67 | |
|  | S2 | 3.228 | T03 | 3.702 | | | 0.474 | | 0.95 | |
|  | S2 | 3.228 | T05 | 3.899 | | | 0.671 | | 0.40 | |
|  | S2 | 3.228 | T06 | 3.899 | | | 0.671 | | 0.90 | |
|  | S2 | 3.228 | T07 | 3.914 | | | 0.686 | | 0.85 | |
|  | S2 | 3.228 | T09 | 3.94 | | | 0.712 | | 0.95 | |
|  | S2 | 3.228 | T12 | 3.998 | | | 0.77 | | 0.39 | |
|  | S2 | 3.228 | T13 | 4.009 | | | 0.781 | | 0.39 | |
|  | S2 | 3.228 | T15 | 4.025 | | | 0.797 | | 2.40 | |
|  | S2 | 3.228 | T18 | 4.047 | | | 0.819 | | 1.29 | |
|  | S2 | 3.228 | T19 | 4.113 | | | 0.885 | | 0.66 | |
| **Two Cl up** | | | | | | | | | | |
| 2x -10 | S1 | 3.189 | T04 | 3.673 | | | 0.484 | | 2.44 | |
|  | S1 | 3.189 | T06 | 3.88 | | | 0.691 | | 3.23 | |
|  | S1 | 3.189 | T07 | 3.916 | | | 0.727 | | 0.94 | |
|  | S1 | 3.189 | T10 | 3.939 | | | 0.75 | | 0.88 | |
|  | S1 | 3.189 | T15 | 4.046 | | | 0.857 | | 0.55 | |
|  | S1 | 3.189 | T16 | 4.046 | | | 0.857 | | 6.55 | |
|  | S1 | 3.189 | T17 | 4.059 | | | 0.87 | | 0.57 | |
|  | S1 | 3.189 | T18 | 4.059 | | | 0.87 | | 3.96 | |
|  | S1 | 3.189 | T19 | 4.117 | | | 0.928 | | 1.79 | |
|  | S2 | 3.19 | T03 | 3.673 | | | 0.483 | | 2.44 | |
|  | S2 | 3.19 | T05 | 3.88 | | | 0.69 | | 3.23 | |
|  | S2 | 3.19 | T08 | 3.916 | | | 0.726 | | 0.93 | |
|  | S2 | 3.19 | T09 | 3.939 | | | 0.749 | | 0.87 | |
|  | S2 | 3.19 | T15 | 4.046 | | | 0.856 | | 6.54 | |
|  | S2 | 3.19 | T16 | 4.046 | | | 0.856 | | 0.55 | |
|  | S2 | 3.19 | T17 | 4.059 | | | 0.869 | | 3.97 | |
|  | S2 | 3.19 | T18 | 4.059 | | | 0.869 | | 0.57 | |
|  | S2 | 3.19 | T20 | 4.117 | | | 0.927 | | 1.78 | |
|  |  |  |  |  | | |  | |  | |
| **Cl···H tetramer** | | | | | | | | | | |
| **Optimal** | 4 | 3.341 | 9 | | 3.707 | 0.366 | | 0.86 | |  |
|  | 4 | 3.341 | 10 | | 3.828 | 0.487 | | 0.39 | |  |
|  | 4 | 3.341 | 11 | | 3.852 | 0.511 | | 0.31 | |  |
|  | 4 | 3.341 | 12 | | 3.86 | 0.519 | | 0.94 | |  |
|  | 4 | 3.341 | 13 | | 3.869 | 0.528 | | 0.4 | |  |
|  | 4 | 3.341 | 14 | | 3.875 | 0.534 | | 0.32 | |  |
|  | 3 | 3.286 | 10 | | 3.828 | 0.542 | | 0.51 | |  |
|  | 4 | 3.341 | 15 | | 3.883 | 0.542 | | 0.58 | |  |
|  | 4 | 3.341 | 16 | | 3.884 | 0.543 | | 0.65 | |  |
|  | 4 | 3.341 | 20 | | 3.905 | 0.564 | | 0.36 | |  |
|  | 3 | 3.286 | 14 | | 3.875 | 0.589 | | 0.66 | |  |
|  | 3 | 3.286 | 15 | | 3.883 | 0.597 | | 0.79 | |  |
|  | 3 | 3.286 | 16 | | 3.884 | 0.598 | | 0.67 | |  |
|  | 4 | 3.341 | 23 | | 3.94 | 0.599 | | 0.53 | |  |
|  | 4 | 3.341 | 26 | | 3.965 | 0.624 | | 3.88 | |  |
|  | 4 | 3.341 | 28 | | 3.984 | 0.643 | | 0.83 | |  |
|  |  |  | **Cl2 to Acceptor** | |  |  | |  | |  |
| +10 | S4 | 3.366 | t05 | | 3.635 | 0.269 | | 0.29 | |  |
|  | S4 | 3.366 | t06 | | 3.651 | 0.285 | | 0.55 | |  |
|  | S4 | 3.366 | t09 | | 3.708 | 0.342 | | 0.24 | |  |
|  | S3 | 3.285 | t05 | | 3.635 | 0.35 | | 0.34 | |  |
|  | S4 | 3.366 | T11 | | 3.855 | 0.489 | | 0.92 | |  |
|  | S4 | 3.366 | T12 | | 3.858 | 0.492 | | 0.92 | |  |
|  | S3 | 3.285 | T10 | | 3.824 | 0.539 | | 0.53 | |  |
|  | S3 | 3.285 | T11 | | 3.855 | 0.57 | | 0.26 | |  |
|  | S4 | 3.366 | T25 | | 3.947 | 0.581 | | 0.24 | |  |
|  | S3 | 3.285 | T13 | | 3.87 | 0.585 | | 0.22 | |  |
|  | S3 | 3.285 | T14 | | 3.875 | 0.59 | | 0.67 | |  |
|  | S4 | 3.366 | T26 | | 3.96 | 0.594 | | 1.45 | |  |
|  | S3 | 3.285 | T15 | | 3.884 | 0.599 | | 0.92 | |  |
|  | S3 | 3.285 | T16 | | 3.885 | 0.6 | | 0.44 | |  |
|  | S4 | 3.366 | T28 | | 3.982 | 0.616 | | 0.27 | |  |
|  |  |  | **Cl2 to Donor** | |  |  | |  | |  |
| -10 | S4 | 3.317 | t05 | | 3.628 | 0.311 | | 1.9 | |  |
|  | S4 | 3.317 | t06 | | 3.648 | 0.331 | | 0.49 | |  |
|  | S3 | 3.285 | t06 | | 3.648 | 0.363 | | 0.35 | |  |
|  | S4 | 3.317 | t09 | | 3.703 | 0.386 | | 1.98 | |  |
|  | S4 | 3.317 | T10 | | 3.825 | 0.508 | | 2.53 | |  |
|  | S4 | 3.317 | T11 | | 3.847 | 0.53 | | 1.14 | |  |
|  | S3 | 3.285 | T10 | | 3.825 | 0.54 | | 0.46 | |  |
|  | S4 | 3.317 | T12 | | 3.862 | 0.545 | | 0.94 | |  |
|  | S4 | 3.317 | T13 | | 3.868 | 0.551 | | 0.37 | |  |
|  | S4 | 3.317 | T14 | | 3.874 | 0.557 | | 1.13 | |  |
|  | S3 | 3.285 | T11 | | 3.847 | 0.562 | | 0.29 | |  |
|  | S4 | 3.317 | T15 | | 3.883 | 0.566 | | 1.51 | |  |
|  | S4 | 3.317 | T16 | | 3.884 | 0.567 | | 0.48 | |  |
|  | S3 | 3.285 | T13 | | 3.868 | 0.583 | | 0.23 | |  |
|  | S4 | 3.317 | T20 | | 3.905 | 0.588 | | 0.78 | |  |
|  | S3 | 3.285 | T14 | | 3.874 | 0.589 | | 0.66 | |  |
|  | S3 | 3.285 | T15 | | 3.883 | 0.598 | | 0.53 | |  |
|  | S3 | 3.285 | T16 | | 3.884 | 0.599 | | 0.91 | |  |
|  | S4 | 3.317 | T21 | | 3.917 | 0.600 | | 0.36 | |  |
|  | S4 | 3.317 | T22 | | 3.936 | 0.619 | | 0.7 | |  |
|  | S4 | 3.317 | T24 | | 3.942 | 0.625 | | 1.45 | |  |
|  | S4 | 3.317 | T25 | | 3.945 | 0.628 | | 0.53 | |  |
|  | S4 | 3.317 | T26 | | 3.964 | 0.647 | | 7.21 | |  |
|  | S4 | 3.317 | T27 | | 3.986 | 0.669 | | 1.35 | |  |
|  | S3 | 3.285 | T26 | | 3.964 | 0.679 | | 0.27 | |  |
|  | S3 | 3.285 | T27 | | 3.986 | 0.701 | | 0.26 | |  |

**Table S9**. Calculated energies for the species with various ∠C-C-Cl [°]

| **Species** | **∠C-C-Cl [**°**]** | **E(Hartree)** | **Δ*E* [eV]** |
| --- | --- | --- | --- |
| **Cl···Cl dimer** | | | |
| optimal | 118 | -3270.473485 | 0.00 |
| 1Cl to A | +10 | -3270.466323 | 0.19 |
| 1Cl to D | -10 | -3270.461700 | 0.32 |
| 2xCl to A | 2x +10 | -3270.459102 | 0.39 |
| 1Cl to A,2 Cl to D | +10,-10 | -3270.454916 | 0.51 |
| 2xCl to D | 2x -10 | -3270.449443 | 0.65 |
| **Cl···H tetramer** | | | |
| optimal | 118 | -6540.926928 | 0.00 |
| Cl to A | +10 | -6540.918912 | 0.22 |
| Cl to D | -10 | -6540.916212 | 0.29 |

In the Cl···Cl dimer, vibrational distortions were analyzed for the two chlorine atoms that form the intermolecular Cl···Cl contact between adjacent monomeric units. According to the changes of Gibbs free energy (Table S9, SI), displacement of one chlorine atom is energetically accessible. Motion toward the acceptor (Δ_A_ = 10°) is the most favorable direction, while motion toward the donor (Δ_D_ = 10°) requires an additional 130 meV. Displacement of both Cl atoms in the same direction (2 x Δ_A_) increases the energy by 200 meV, whereas opposite displacements require 310 meV (Δ_A_ & Δ_D_) or 460 meV (2 x Δ_D_), indicating that cooperative vibrations of both chlorines are unlikely at room temperature (Table S9, SI).

Within the most probable single-atom motions, both directions of distortion break the degeneracy of the two monomeric units forming the Cl···Cl dimer, leading to an energy splitting of 23 meV (Δ_D_) and 28 meV (Δ_A_) between the S_1_ and S_2_ states.

Importantly, such Cl motions strongly enhance SOC with the ^3^LE states (Figure 7E, maintext). For example, for the lowest energy ^3^LE_A_ state (T_4_), the ⟨^1^CT∣Ĥ_SO_∣^3^LE_A_⟩ value increases from 0.45 cm^-1^ (equilibrium) to 0.94 cm^-1^ (Δ_A_) and up to 2.3 cm^-1^ (Δ_D_). Several additional spin-flip channels require high energies but illustrate well the role of Cl vibrations. For example, ⟨S_2_∣Ĥ_SO_∣T_5_⟩: 1.0 cm^-1^ (Δ_A_) and 3.1 cm^-1^ (Δ_D_); ⟨S_2_∣Ĥ_SO_∣T_15_⟩: 2.4 cm^-1^ (Δ_A_) and 4.1 cm^-1^ (Δ_D_); ⟨S_1_∣Ĥ_SO_∣T_16_⟩: 3.1 cm^-1^ (Δ_A_) and 5.7 cm^-1^ (Δ_D_). Overall, the Cl motion toward the acceptor activates a number of moderately coupled transitions, whereas motion toward the donor produces the strongest SOC enhancement, providing the most efficient spin-flip channels.

In the Cl···H tetramer, the analyzed chlorine atom interacts with three neighboring molecules through weak Cl···H–C contacts. Distortions of its ∠C–C–Cl valence angle reveal again that the Δ_A_ motion is slightly more thermodynamically favorable, while the Δ_D_ motion requires an additional 73 meV, which is less than in the Cl···Cl dimer, suggesting greater vibrational flexibility of this site. The relevant singlet state, S_4_, localized on the perturbed molecule (see Figure S9, SI), participates in several low-gap S–T transitions whose SOC values are strongly modulated by chlorine motion. For the Δ_A_ distortion, the SOC for the lowest-energy channels (S_4_-T_6_, S_4_-T_11_) increases moderately to 0.55–0.92 cm^-1^, while Δ_D_ distortions produce much stronger enhancement, reaching 1.9 cm^-1^ for S_4_-T_5_ and peaking at 7.2 cm^-1^ for S_4_-T_26_ (Figure 7F, maintext**)**.

**Figure S9.** Molecular orbitals and natural transition orbitals

**DMAC-TRZ**

**DMAC-diF-TRZ**

**Analyses**

**^1^H-NMR of DMAC-diCl-TRZ**

**^13^C-NMR of DMAC-diCl-TRZ**

**^1^H-NMR of DMAC-diF-TRZ**

**^13^C-NMR of DMAC-diF-TRZ**

**^1^H and ^13^C-NMR of triF-TRZ**

**^1^H NMR of DMAC-diClPhBr**

**^13^C-NMR of DMAC-diClPhBr**

**^1^H NMR of DMAC-diClPhBpin**

**^13^C-NMR of DMAC-diClPhBpin**

**References**

1. [S] CrysAlis CCD and CrysAlis RED, Version 1.171.36.24; Oxford Diffraction Ltd.: Yarnton, UK, **2012**
2. [S] G. M. Sheldrick, *Acta Crystallogr., Sect. C: Struct. Chem.* **2015**, *71*, 3–8 https://doi.org/10.1107/S2053229614024218
3. [S] C. K. Johnson, ORTEP II, Report ORNL-5138; Oak Ridge National Laboratory: Oak Ridge, TN, USA, **1976**
4. [S] C. F. Macrae, I. Sovago, S. J. Cottrell, P. T. A. Galek, P. McCabe, E. Pidcock, M. Platings, G. P. Shields, J. S. Stevens, M. Towler, P. A. Wood, *J. Appl. Crystallogr.* **2020**, *53*, 226–235. https://doi.org/10.1107/S1600576719014092
5. [S] A. A. Kubicki, P. Bojarski, M. Grinberg, M. Sadownik, and B. Kukliński, *Optics Communications* **2006**, 263: 275–280, https://doi.org/ 10.1016/j.optcom.2006.01.043
6. [S] F. Neese, *WIREs Comput. Mol. Sci.* **2025**, *15*, e70019. https://doi.org/10.1002/wcms.70019
7. [S] T. Yanai, D. P. Tew, N. C. Handy, Chem. *Phys. Lett.* **2004**, *393*, 51–57. DOI: 10.1016/j.cplett.2004.06.011
8. [S] Y. Zhao, D. G. Truhlar, *Theor. Chem. Acc.* **2008**, *120*, 215–241. https://doi.org/10.1007/s00214-007-0310-x
9. [S] L. M. Bollinger, G. E. Thomas, *Rev. Sci. Instrum.* **1961**, *32*, 1044–1050. https://doi.org/10.1063/1.1717610
10. [] T.-A. Lin, T. Chatterjee, W.-L. Tsai, W.-K. Lee, M.-J. Wu, M. Jiao, K.-C. Pan, C.-L. Yi, C.-L. Chung, K.-T. Wong, C.-C. Wu, *Adv. Mater.* **2016**, *28*, 6976–6983. DOI: 10.1002/adma.201601675
11. [S] M. J. Berger, J. H. Hubbell, NIST Standard Reference Database 8: XCOM: Photon Cross Sections Database, National Institute of Standards and Technology, Gaithersburg, MD, 1990
12. [S] C. T. Chantler, *J. Phys. Chem. Ref. Data* **1995**, *24*, 71–643. https://doi.org/10.1063/1.555974
